# Assessment of the ground vibration during blasting in mining projects using different computational approaches

**DOI:** 10.1038/s41598-023-46064-5

**Published:** 2023-10-30

**Authors:** Shahab Hosseini, Jitendra Khatti, Blessing Olamide Taiwo, Yewuhalashet Fissha, Kamaldeep Singh Grover, Hajime Ikeda, Mukesh Pushkarna, Milkias Berhanu, Mujahid Ali

**Affiliations:** 1 Faculty of Engineering, Tarbiat Modares University, Tehran, Iran; 2 Department of Civil Engineering, Rajasthan Technical University, Kota, Rajasthan India; 3 Department of Mining Engineering, Federal University of Technology, Akure, HNF Global Resources Limited, Akoko Edo, Nigeria; 4 Department of Geosciences, Geotechnology, and Materials Engineering for Resources, Graduate School of International Resource Sciences, Akita University, Akita, 0108502 Japan; 5 Department of Electrical Engineering, GLA University Mathura, Mathura, India; 6 Department of Civil Engineering, Adama Science and Technology University, Adama, Ethiopia; 7 Department of Civil Engineering, Silesian University of Technology, Gliwice, Poland

**Keywords:** Engineering, Mathematics and computing, Physics

## Abstract

The investigation compares the conventional, advanced machine, deep, and hybrid learning models to introduce an optimum computational model to assess the ground vibrations during blasting in mining projects. The long short-term memory (LSTM), artificial neural network (ANN), least square support vector machine (LSSVM), ensemble tree (ET), decision tree (DT), Gaussian process regression (GPR), support vector machine (SVM), and multilinear regression (MLR) models are employed using 162 data points. For the first time, the blackhole-optimized LSTM model has been used to predict the ground vibrations during blasting. Fifteen performance metrics have been implemented to measure the prediction capabilities of computational models. The study concludes that the blackhole optimized-LSTM model PPV11 is highly capable of predicting ground vibration. Model PPV11 has assessed ground vibrations with RMSE = 0.0181 mm/s, MAE = 0.0067 mm/s, R = 0.9951, a20 = 96.88, IOA = 0.9719, IOS = 0.0356 in testing. Furthermore, this study reveals that the prediction accuracy of hybrid models is less affected by multicollinearity because of the optimization algorithm. The external cross-validation and literature validation confirm the prediction capabilities of model PPV11. The ANOVA and Z tests reject the null hypothesis for actual ground vibration, and the Anderson–Darling test rejects the null hypothesis for predicted ground vibration. This study also concludes that the GPR and LSSVM models overfit because of moderate to problematic multicollinearity in assessing ground vibration during blasting.

## Introduction

**Figure 1:**
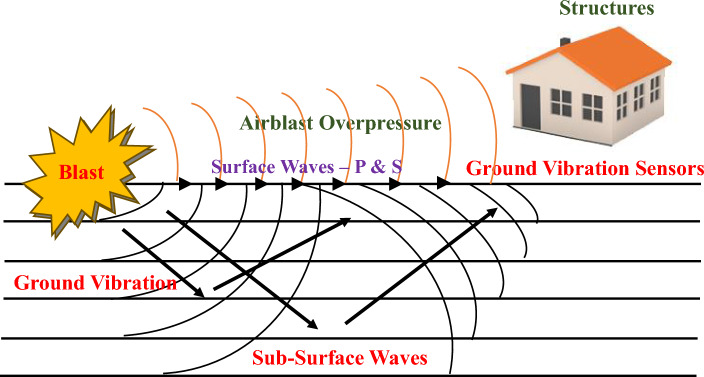
Ground vibration during blasting^2^.

The detonation of the rock mass during the blasting process causes ground vibration. An explosive charge is inserted into the blast hole in order to explode and shatter rocks. Due to the quick rock acceleration after the blast hole is detonated, significant dynamic stresses are created. The transmission of strain waves by rock mass results in the generation of a wave motion. These strain waves' strain energy causes the rock mass to break apart through a variety of breakage mechanisms, including crushing, radial cracking, and reflection breakage in the presence of a free face. A volume of rock is permanently distorted inside the crushed zone and radial fracture zone. Beyond the fragmentation zone, where there is no permanent rock mass deformation due to stress waves, strain waves propagate through the medium as elastic waves, oscillating the particles they pass through^1^. Figure 1 depicts the ground vibration during blasting.

Rock blasting is one of the most common and cost-efficient rock excavation techniques in mining and civil engineering projects^3,4^. Rock blasting is a prominent mining technique for metal and non-metal resources, such as hard rock mining excavations and quarrying. According to^5^, around 30% of the energy from the whole explosive is successfully utilized to break up the rock during rock blasting, while the remaining energy is wasted either through blasting vibration, fly rock, back break, or air overpressure, etc. One of the most severe environmental impacts of blasting is ground vibration, which also causes issues for individuals in the proximity of the blasting zone^6^. The release of energy during the explosion is what generates ground vibration, and the strength of the vibration is determined by several factors, including the quantity of explosive used, the type of rock blasted, and the distance from the site of the blast to the monitoring station making the optimization a bit challenging without good simulation. According to Lawal and Idris^7^, the blast-induced ground vibration's intensity is associated with the controlled and uncontrollable blasting parameters. Fattahi and Hasanipanah^8^ assessed the ground vibration using an optimized relevance vector machine with a performance (R) of 0.915.

During the release of waves from explosive detonation, mine blasting activities cause ground and structures to vibrate. Monitoring and regulating blast-induced ground vibration through predicting peak particle velocity (PPV) has been a concern of blasting engineers for decades. Singh et al.^9^ noted that the frequency of blast-induced vibration plays a crucial role in destroying structures near a mine. The impact of blast-induced vibrations on surrounding structures, sensitive equipment, and people in urban environments is a crucial aspect of a sustainable mining project. Variables such as mining technique, blast design parameters, explosive type, usage and quantity, rock medium, heterogeneity of rock deposit at the site, distance from the source, characteristics of wave propagation at a site, dynamic characteristics of soil and rocks, response characteristics of existing rock mass fractures, and so on all contribute to the intensity of vibrations on the ground caused by mining operations^10–12^. Peak particle velocity is produced during tensile slabbing by superimposing the induced blast waves from each hole beyond the crushing zone. According to Garai et al.^13^, the size of the resulting peak particle velocity is determined mainly by the geometry of the drill hole. Since the superimposition angle of the wave is affected by the design orientation sequence of the hole blasts performed on-site, there is an extensive range in the resulting PPV, which can be estimated using machine-learning techniques. Nateghi^10^ explained that many of these parameters, including rocks' geological and geotechnical conditions, cannot be altered. However, the amount of explosives detonated per delay (as a controllable parameter) can be estimated using an empirical formula that can account for geological and blast design conditions^10^. Valdivia et al.^14^ and Rossmanith et al.^15^ demonstrated the significance of a number of variables whose modifications can affect vibration reduction and blasting operation enhancement. Several literature works revealed a plethora of papers on blast-induced effects, most of which described ground vibration as a difficult byproduct of explosive use in the mine as a technique for rock excavation. Explosive-generated waves dissipate with distance relatively regularly, making them predictably accurate and enabling the regulation of blasting vibrations via mathematical expressions or machine learning soft computing.

The number of explosives used, the distance between the blast face and the monitoring point, and the geological and geotechnical conditions of the rock units in the excavation area are all directly related to ground vibration^16,17^. Bui et al.^18^ examined the effects of blasting in a quarry mine in Vietnam using an uncrewed aerial vehicle. The collected database was utilized to create a novel model of hybrid intelligence. Their discovery demonstrates that the negative effects of blast-induced ground vibration can be mitigated using a novel hybrid model. Lawal et al.^19^ examined the Gene expression programming (GEP), adaptive neuro-fuzzy inference system (ANFIS), and sine cosine algorithm-optimized artificial neural network (SCA-ANN) models to predict blast-initiated ground vibration in five granite quarries. For modelling the blast peak particle velocity, 100 datasets and eight input parameters (distance from the point of blasting to the point of measurement, weight of charge per delay, rock density, and Schmidt rebound hardness) were considered. The deformation behavior of rock during micro-fracturing by blast waves significantly influences the propagation and utilization of explosive energy. The study considered new input parameters for the ground vibration model to incorporate the influence of deformation. Duan et al.^20^ developed an LSSVM model for estimating greater heating values. Rad et al.^21^ predicted blast-induced fly-rock in the Gole-E-Gohar iron mine in Iran using the LSSVM approach. Huang et al.^22^ estimated coal thickness using the LSTM method to improve coal mining safety in the Ordos Basin. Zhang used LSTM to predict landslide failure at Fengning Landslide to overcome the difficulty in predicting stepped landslide deformation with short-term influence. Gomilanovic et al.^23^ predicted the availability of continuous systems in open pit mines using LSTM. To better comprehend the blast-generated vibration full waveform at Hongtuoshan Copper Mine, Wang et al.^24^ developed a new full waveform prediction model using LSTM. These applications demonstrate the applicability of artificial intelligence techniques to various geo-engineering and mining problems, including ground vibration prediction. Table 1 displays the summary of the literature survey to estimate the ground vibration level during the blasting.

**Table 1:** Details of AI models available in the literature review.

References	AI technique	Number of datapoints	Details of input variables		R^2^ Test
			Variables used	Nos	
Nguyen et al.^25^	SSO-ELM	216	B/S, DI, ST, MC, PF, HD, RQD, N, SD	9	0.91
Ragam et al.^26^	XGBoost-RF	121	DI, ST, MC, PF, HD	5	0.95
Zhang et al.^27^	**RF**, CART, CHAID	102	DI, MC	2	0.94
Zhou et al.^28^	RF	102	DI, MC, PF, B, S, HD	6	0.93
Huang et al.^29^	FA-ANN	88	DI, MC, B, S, N	5	0.91
Zhou et al.^30^	GEP-MC	102	B/S, DI, ST, MC, PF, HD	6	0.91
Nguyen et al.^11^	SVR-GA	125	DI, MC	2	0.99
Nguyen et al.^12^	HKM-CA	136	DI, MC	2	0.99
Armaghani et al.^31^	ICA	73	B, S, ST, D, HD, PF, MC, DI	8	0.95
Hasanipanah et al.^32^	CART	86	DI, MC	2	0.95
Shahnazar et al.^33^	PSO-ANFIS	81	–	–	0.98
Ghoraba et al.^34^	ANN, **ANFIS**	115	DI, MC	2	0.95
Shirani Faradonbeh et al.^35^	GEP	102	DI, MC	2	0.88
Hajihassani et al.^36^	ICA-ANN	95	MC, DI, TC	3	0.98
Dindarloo^37^	SVM	100	S, B, ST, PF, MC, D, N, RD, SD	9	0.99
Hajihassani et al.^38^	PSO-ANN	88	BS, ST, PF, MC, DI, Vp, E	7	0.89
Hasanipanah et al.^39^	SVM	80	RD, E, UCS, TS, Js, B, S, HD/B, SC, ST, DPR, DI	12	0.96
Armaghani et al.^6^	ANFIS	109	BS, MC, HD, ST, SD, DI, PF, RQD	8	0.97
Armaghani et al.^40^	PSO-ANN	44	B, S, ST, N, MC, DI	6	0.94
Ghasemi et al.^41^	FIS	120	DI, MC	2	0.95
Monjezi et al.^42^	ANN	20	–	–	0.93
Mohamadnejad et al.^43^	**SVM**, ANN	37	DI, MC	2	0.89
Monjezi et al.^44^	ANN	182	CD, DI, ST, HD	4	0.95
Mohamed^45^	**ANN**, FIS	162	DI, MC	2	0.94
Khandelwal et al.^1^	ANN	130	–	–	0.92
Fişne et al.^46^	FIS	33	DI, MC	2	0.92
Iphar et al.^47^	ANFIS	42	DI, CD	2	0.99
Singh and Singh^48^	ANN	–	D, N, HD, B, S, ST, MC, HDI, RDI	9	0.82

*Bold values represent the best soft computing model in the reported study.

### Gap identification

A thorough study of the published research on assessing ground vibration using soft computing approaches reveals that the soft computing approaches are highly capable of predicting ground vibration for mining projects. In the published studies, the researchers and scientists employed various computational approaches using different databases. However, it is already known that the prediction capabilities of soft computing models are based on the nature of databases. Therefore, the optimum performance model in predicting ground vibration is still questionable. Also, the least square support vector machine (LSSVM), ensemble tree (ET), decision tree (DT), gaussian process regression (GPR), support vector machine (SVM), multilinear regression (MLR), artificial neural network (ANN), and long short-term memory (LSTM) models have not been employed for assessing the ground vibrations utilizing a common database. Furthermore, the blackhole optimization algorithm has not been implemented with the LSTM (LSTM-BA) approach and compared to LSSVM, ET, DT, GPR, ET, DT, SVM, MLR, and ANN models in predicting ground vibrations. The Gaussian, polynomial, and linear kernels have not been implemented with the LSSVM model and compared in predicting ground vibration. Multicollinearity is a significant factor for the model's accuracy, which was not studied in predicting ground vibration. In addition, statistical tests, such as Anderson–Darling, Z, and ANOVA, for determining the quality of the database and selection of the hypothesis have not been performed in the reported studies.

### Research objectives

This research has been conducted to find the optimum performance model for predicting ground vibration in mining projects. For that aim, the MLR, SVM GPR, DT, ET, LSSVM (gaussian, polynomial, and linear), Bayesian regularization-optimized artificial neural network (ANN-BR), LSTM, and LSTM-BA models have been employed using a common database, and performances have been analyzed. In addition, the database multicollinearity has been computed, and its impact has been investigated on predictive accuracy, performance, and overfitting of models. Moreover, three new index parameters, namely, scatter index (IOS), a20, and agreement index (IOA), have been introduced to measure the computational accuracy of employed models.

### Research significance

Determining ground vibration during blasting is time-consuming and arduous. Therefore, a new methodology and experimental setup are required to minimize the impact during blasting. The present research introduces a new methodology associated with artificial intelligence, which is less time-consuming, accurate, and helpful to mining engineers/ designers.

## Research methodology

**Figure 2:**
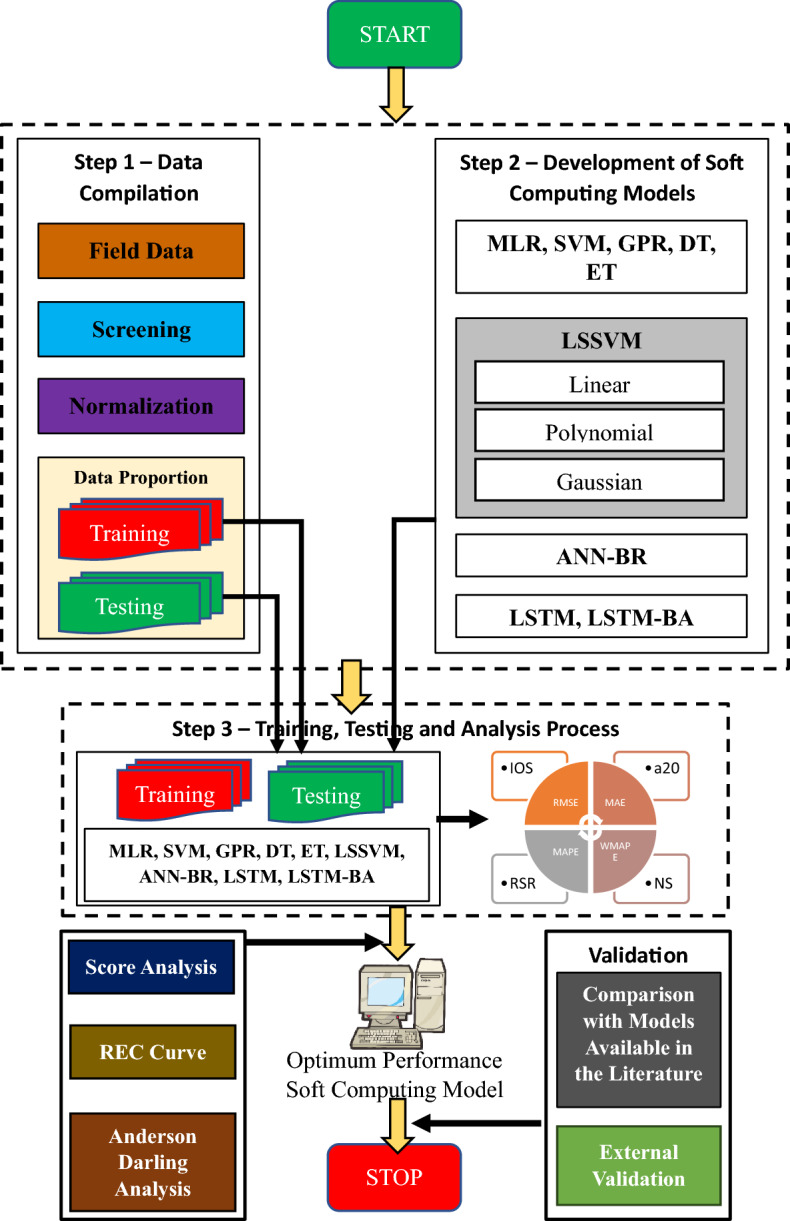
Illustration of a flow chart of the present research.

This study has been mapped to identify the optimum performance model for predicting ground vibration (peak particle velocity-PPV). For this aim, MLR, SVM, GPR, DT, ET, LSSVM (linear, polynomial, and Gaussian kernel-based), ANN-BR, LSTM, and LSTM-BA models have been employed, trained, tested, and analyzed. The PPV database has been collected from mines in southwestern Zanjan province in Iran. The database consists of n, B/De, H/B, B (m), S (m), SD (m/kg^0.5^), and PPV (mm/sec) results of 162 boreholes. For training and testing, 80% and 20% of 162 data points have been chosen randomly. RMSE, MAE, MAPE, WMAPE, VAF, NS, NMBE, RSR, R, R^2^, LMI, a20-index, IOS, and IOA statistical parameters have computed the model's predictive capabilities. In addition, the score and regression error characteristics (REC) analyses have been performed to find the optimum performance model for assessing ground vibrations in blasting. Furthermore, the optimum performance model has been validated by comparing the performance of models available in the literature. Moreover, an external validation has been conducted to check the generalizability of the models. Also, the sensitivity, hypothesis (ANOVA and Z test), and multicollinearity analyses have been performed in this research. Figure 2 depicts the execution of the present investigation.

## Data analysis and soft computing approaches

### Data collection and data analysis

**Figure 3:**
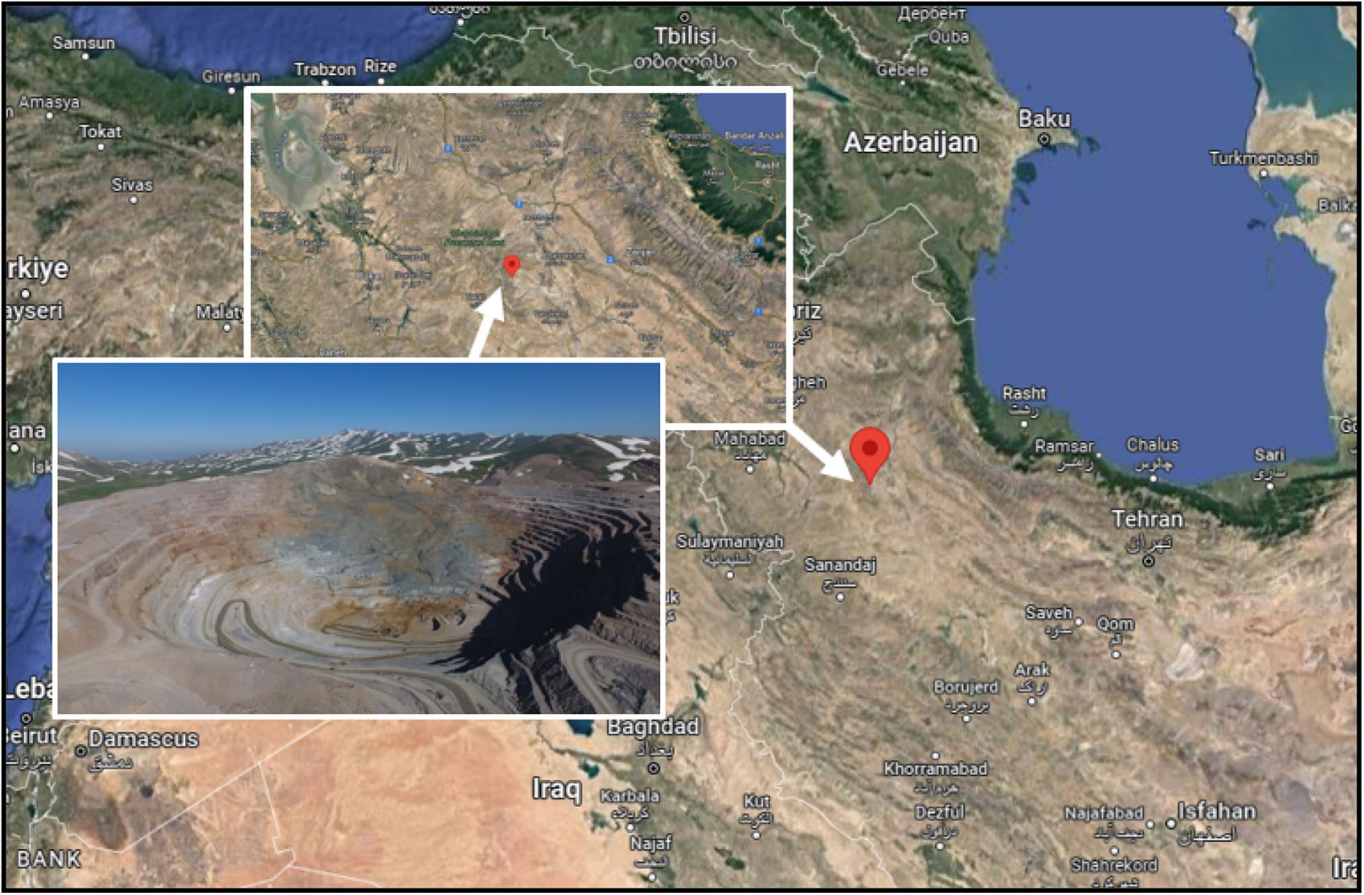
Geographical location of mine (130 km from southwestern Zanjan province in Iran).

The PPV (ground vibration) database has been accumulated from the biggest lead–zinc surface mine in Midleast. The geographical location of this mine is approximately 130 km from southwestern Zanjan province in Iran. The longitude, latitude, and altitude above sea level of the Anguran mine are 478225 E, 368433 N, and 2954 m (refer Fig. 3). Since 1940, excavation has been placed at the Anguran mine, which is Iran's biggest and oldest lead and zinc mine. The Anguran mine is one case of faulty management, and as a result, the mine's northern and western sides have been plagued by many instability problems. The location is a portion of the Oroumieh-Poldokhtar region, which stretches between the Zagros belt and the central Iranian region. The mine is located in the centre of a turning anticline, and the predominant rock in the mining region is a metamorphic limestone that contains graphite. The influence of folds is less noticeable after one moves through the carbonaceous portion of the area and into the metabasin stones to the northwest. Various volcanoes, sediment, and metamorphic rocks compose the study region. There is significant evidence in the region of volcanic and plutonic activities. These are the key occurrences of metamorphic rocks and hydrothermal incursion that led to the formation of the various mineral veins at the mine. The deposit's comprehensive geologic resources are around 25 Mt, while its proved reserves are around 12 Mt, with average zinc and lead grades of 27.87% and 4.26%, respectively.

The Anguran mine's blasted benches are conducted by blast holes with a diameter of 5 inches, explosive materials with a specific gravity of 0.85 g/cm^3^ (ANFO), vertical blast holes, and a delay timing of 3–5 s. To make ANFO, ammonium nitrate (in powder or tablet form) should be mixed with gasoline or oil in a 94.5–5.5% ratio. Peak particle velocity, which leads to damage to the pit walls during blast rounds, is the most challenging issue (refer Fig. 2b,c). It is widely accepted that the blasting pattern design factors affect PPV. After examining these factors, measurements of the burden, spacing, hole depth, charge per delay, sub-drilling, and PPV—the variables that have the most significant impact on back-break and fragmentation—were performed at the Anguran mine by monitoring the 162 different blasting rounds.

**Figure 4:**
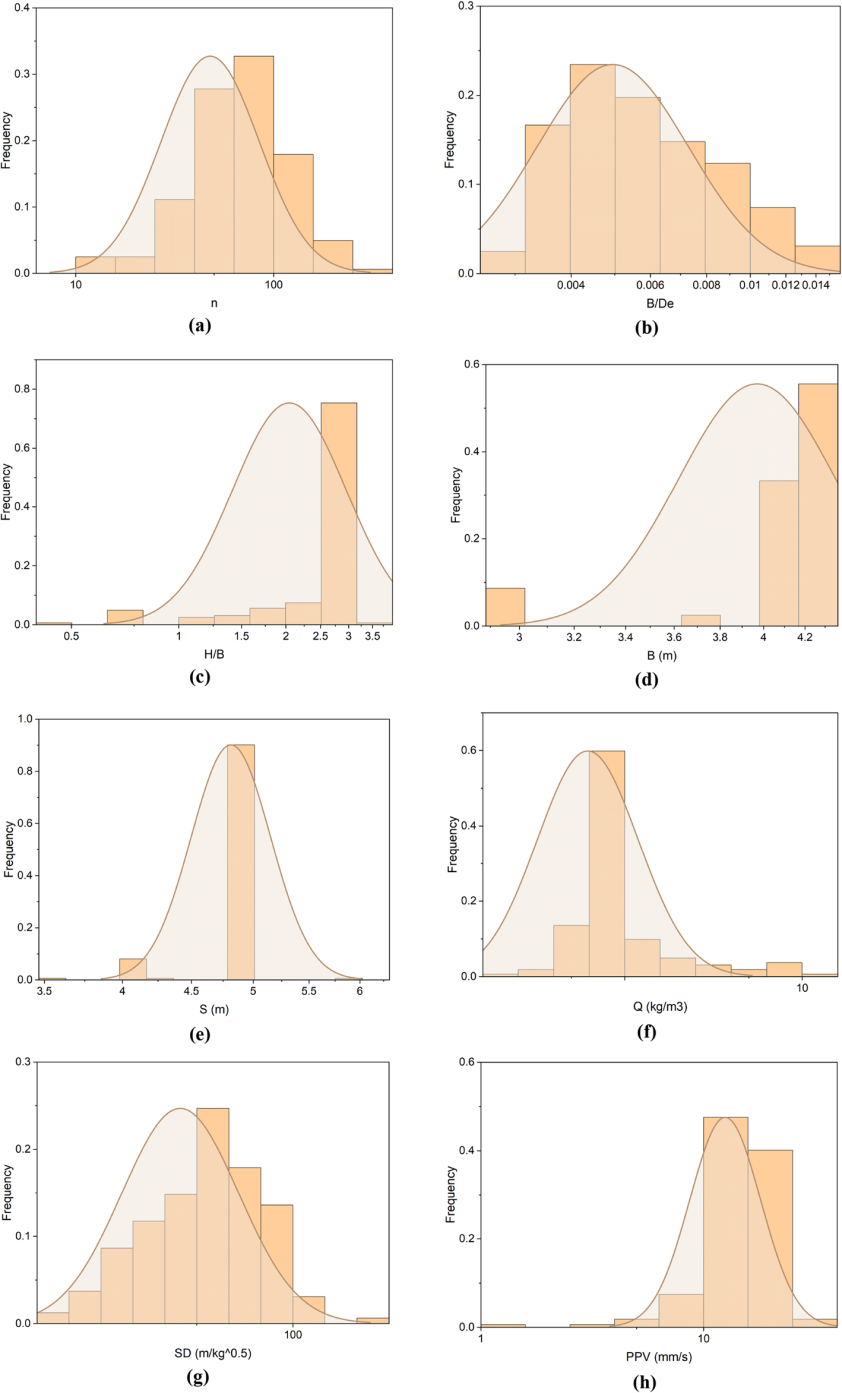
Illustration of the frequency distribution of variables: (**a**) n, (**b**) B/De, (**c**) H/B, (**d**) B, (**e**) S, (**f**) Q, (**g**) SD, and (**h**) PPV.

Each database has numerous data points spread across numerous rows and columns, which makes it challenging to comprehend. Descriptive statistics are thus generated for the database. In the present research, descriptive statistical parameters, mean, SE mean, StDev, variance, coefficient of variance, minimum, Q1, median, Q3, maximum, IQR, skewness, kurtosis, and MSSD have been calculated for overall, training, testing databases, as mentioned in Table A (refer Appendix). Table A demonstrates that the overall database contains a number of drilling holes (n), burden-to-diameter ratio (B/De), stiffness ratio (H/B), burden (B in meters), spacing (S in meters), explosive weight (Q in kg/m^3^), scaling distance (SD in m/kg^0.5^), and peak particle velocity (PPV in mm/s) in the range of 10.0–323.0, 0.003–0.014, 0.476–3.833, 3.0–4.2, 3.5–6.0, 0.229–12.082 kg/m^3^, 16.6–160.42 m/kg^0.5^, and 1.135–28.197 mm/s. Before utilizing the database for training and testing purposes, the database has been preprocessed, and missing data and outliers have been removed and normalized by the min–max normalization function $= \left(x-min\right)/\left(\mathrm{max}- min\right)$, where x is the actual value. As a result, Fig. 4 depicts the frequency distribution of the database's n, B/De, H/B, B, S, Q, SD, and PPV variables.

#### Coefficient of correlation

**Figure 5:**
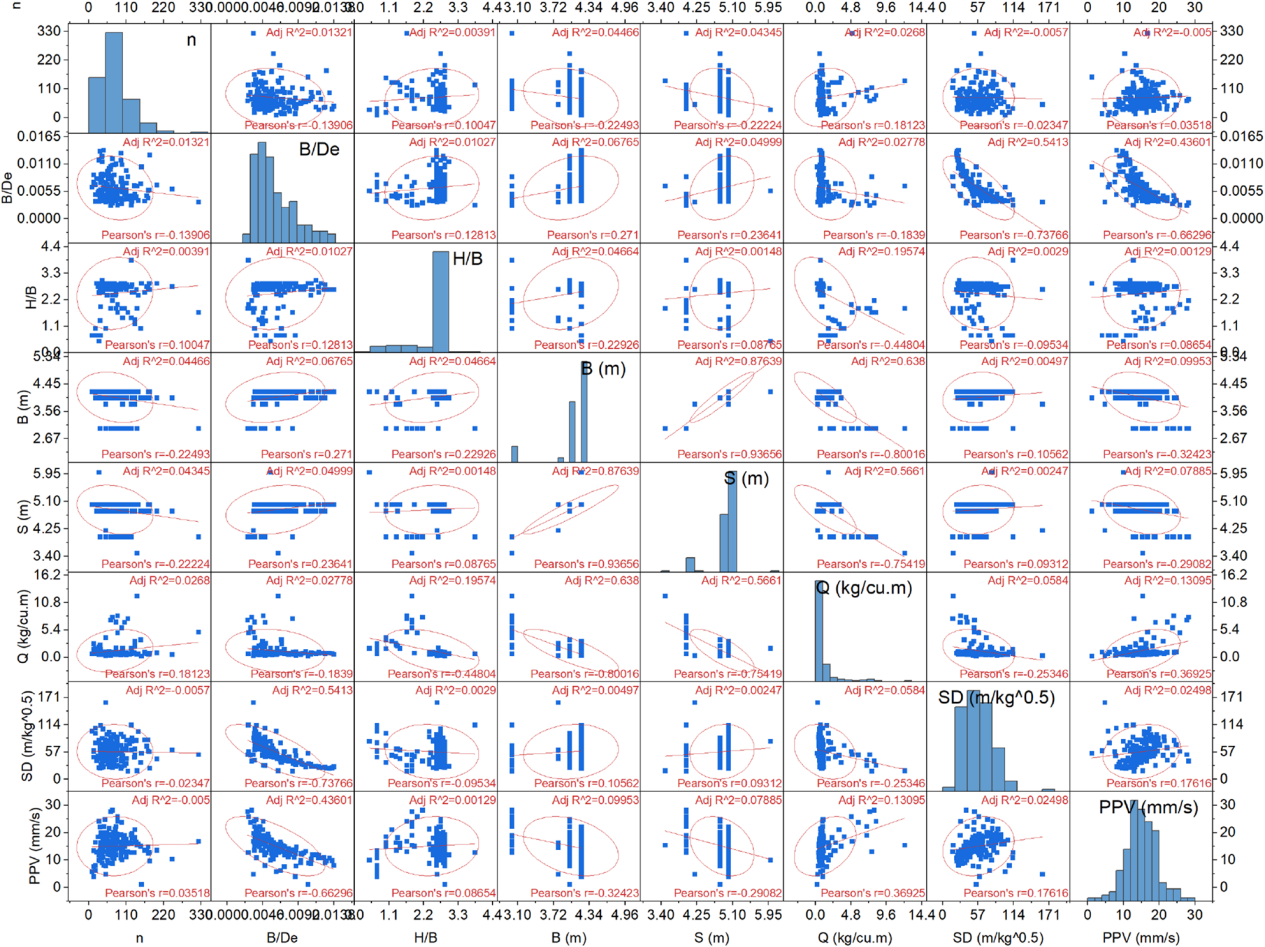
Illustration of the correlation coefficient.

In the database, several columns are available, and each column has a relationship with other columns. Using the Pearson product-moment correlation coefficient, this relationship is identified. The two databases' correlation coefficients range (between ±) from 0.0 to 0.20, 0.21 to 0.40, 0.60 to 0.80, and 0.81 to 1.0, respectively, show extremely strong, strong, moderate, weak, and no association^49^. Figure 5 illustrates the relationship between variables present in the database in terms of the correlation coefficient.

Figure 5 shows that the n variable has no to weak correlation with B/De (− 0.1391), B (− 0.2249), S (− 0.2222), and SD (− 0.0235), negatively. The negative correlation means a value increases with a decrease. The burden-to-diameter ratio (B/De) also weakly correlates with H/B (0.1281), B (0.2710), and S (0.2364) positively. It has also been observed that the B/De moderately correlates with SD (− 0.7377) and PPV (− 0.6630). Furthermore, the stiffness ratio (H/B) no to weakly correlates with B (0.2293), S (0.0877), SD (− 0.0953), and PPV (0.0865). Also, the stiffness ratio moderately correlates with Q (− 0.4480). Burden (B) and spacing (S) strongly correlate with Q (− 0.8002 for B and − 0.7542 for S). The explosive weight (Q) weakly correlates with SD (− 0.2535) and PPV (0.3693). The correlation coefficient method also determines the multicollinearity of variables in the database. The correlation coefficient of n shows multicollinearity with B (− 0.2249) and S (− 0.2222), which may affect the soft computing models' performance, accuracy, and overfitting in predicting the ground vibrations during blasting. The actual multicollinearity level of input variables has been calculated using the variance inflation factor (VIF), discussed in the next section.

#### Multicollinearity analysis

Multicollinearity is a phenomenon that can affect the reliability of statistical inferences in regression analysis when there is a high correlation between independent variables. To address multicollinearity, researchers often use the variance inflation factor (VIF), which measures the extent to which the variance of the estimated regression coefficient is increased. Several researchers have suggested different multicollinearity levels in terms of variance inflation factor (VIF = $1/(1-{R}^{2})$). Khatti and Grover^49^ have proposed different multicollinearity levels. In the present research, the variance inflation factor (VIF) has been calculated for the input variables, i.e., number of drilling holes, burden-to-diameter ratio, stiffness ratio, burden, spacing, explosive weight, scaling distance, and peak particle velocity (ground vibrations), as mentioned in Table 2.

**Table 2:** Results obtained from the multicollinearity analysis.

Statistical parameters	Intercept	n	B/De	H/B	B	S	Q	SD
Coefficients	5.6651	− 0.0181	− 1855.88	2.7361	− 0.5117	4.4838	1.1532	− 0.0970
Standard error	6.4870	0.0041	132.67	0.4243	1.7689	1.9047	0.2432	0.0157
*t* Stat	0.8733	− 4.4261	− 13.99	6.4489	− 0.2893	2.3541	4.7421	− 6.1741
*P*-value	0.3839	0.0000	0.00	0.0000	0.7727	0.0198	0.0000	0.0000
Lower 95%	− 7.1499	− 0.0262	− 2117.96	1.8980	− 4.0061	0.7211	0.6728	− 0.1280
Upper 95%	18.4802	− 0.0100	− 1593.80	3.5743	2.9826	8.2465	1.6336	− 0.0660
R^2^	–	0.0955	0.7373	0.5302	0.9106	0.9101	0.8180	0.7667
VIF	–	1.1056	3.8064	2.1286	11.1856	11.1268	5.4952	4.2861
Multicollinearity Level		Weak	Considerable	Considerable	Problematic	Problematic	Moderate	Considerable

Table 2 demonstrates that (i) the number of drilling holes (n) has a weak multicollinearity level (0 < VIF ≤ 2.5); (ii) burden-to-diameter ratio (B/De), stiffness ratio (H/B), and scaling distance (SD) have considerable multicollinearity level (2.5 < VIF ≤ 5.0); (iii) explosive weight (Q) has moderate multicollinearity level (5.0 < VIF ≤ 10.0); and (iv) burden (B) and spacing (S) have problematic multicollinearity level (10.0 < VIF).

#### Hypothesis analysis

In statistics, parametric and non-parametric tests are performed to recognize the hypothesis analysis. This study uses parametric statistical tests, namely ANOVA and Z-tests, to identify the hypothesis. The following statements have been drawn to identify the research hypothesis.

i. All independent variables do not affect peak particle velocity (ground vibration) prediction equally.
ii. The stiffness and burden-to-diameter ratios do not correlate highly with ground vibration during blasting.
iii. The explosive weight and burden are not highly dependent on each other during blasting.

The analysis of variance (ANOVA) analyses the amount of variation within each sample concerning the amount of variance between samples to test for variations in population mean values. The results obtained from the ANOVA analysis are given in Table B (refer Appendix). Table B illustrates that (i) the value of F (which is the state value) is higher than F crit (F critical) and (ii) the value of p is lower than the significance value, i.e., 0.05, for each input variable. Hence, the present research **rejects** the **NULL HYPOTHESIS** in predicting the ground vibration during blasting. Furthermore, the Z-test has been performed to select the hypothesis. When the variances are known, and the sample size is large, a z-test is a statistical study to determine if two population means differ. When the z-statistic has a normal distribution, a hypothesis test is referred to as a z-test. Table C (refer Appendix) contains the results obtained from the Z-test. Table C demonstrates that each input variable satisfies the statistical clause for selecting the research hypothesis, i.e., z critical one tail < z critical two tail; p-value (one & two tail) < 0.05. Hence, the present research **accepts** the **RESEARCH HYPOTHESIS** for the present research.

### Applied soft computing approaches

This research has employed MLR, LSSVM, SVM, GPR, DT, ET, ANN, and LSTM models to assess the ground vibrations during blasting in a mining project. These models have been configured by analyzing the published research of Taiwo et al.^50,51^, Hosseini et al.^52,53^, Wang et al.^54^, Khatti and Grover^55–58^.

#### Multiple linear regression (MLR)

Several writers have employed the MVR modelling approach, including Taiwo et al.^4^ for fragmentation size prediction, Al-Bakri and Sazid^59^ for blast-induced impact, and Tella et al.^60^ for dust particle matter prediction. The approach of multivariate regression modeling is utilized to build multiple variable equations and provide estimates for diverse engineering challenges^61^. Numerous writers have utilized this approach to build equations that respond to the influence of the dependent variable on the independent variable^62–64^. The general mathematical statement for multiple inputs and one output is illustrated in Eq. (1).

1 $$ Y = b_{0} + b_{1} X_{1} + b_{2} X_{2} + b_{3} X_{3} + \cdots \cdots \cdots \cdots \cdots + b_{n} X_{n} $$

where b is constant, X_i_ (i.e., X_1_ = n, X_2_ = $B/{D}_{e}$, X_3_ = $H/B$, X_4_ = $B$, X_5_ = $S$, X_6_ = $Q$, X_7_ = $SD$) and Y (= PPV) are input and output variables, respectively. The MVR model proposed in this study has seven independent variables: blast hole number, burden, spacing, stiffness ratio, powder factor, burden-to-diameter ratio, and brittleness ratio, and one dependent variable (Peak particle velocity). The developed MLR model (R^2^ = 0.76) is present in Eq. (2) (derived in the training phase) for this study's PPV prediction.

2 $$ \begin{aligned} PPV & = 3.7728 - 0.01593*n - 1800.96*\frac{B}{{D_{e} }} + 2.9482*\frac{H}{B} - 0.9614*B \\ & \quad + 4.9613*S + 1.2098*Q - 0.0922*SD \\ \end{aligned} $$

#### Least-square support vector machine (LSSVM)

LSSVM is a sophisticated machine-learning technique considered an advanced version of the well-known support vector machine (SVM) approach. It employs converting a quadratic programming problem into a linear equation problem. The stages for the computation of an output "${y}_{i}$" with input vector ϵ R^n^| using LSSVM with {(${x}_{i},{y}_{i}$)|i = 1, …, l} data sample points for training are explained in Eqs. (3–8). The model regression optimization problem is represented by Eq. (3).

LSSVM modeling is used to provide appropriate solution adjustment to the computational complexity working problem related to support vector models^65,66^. The work of Razavi et al.^67^ focuses on the use of LS-SVM and ANFIS in model construction for evaluating the thermal conductivity increase of metal and metal oxide-based nanofluids. According to their findings, the least square support vector machine algorithm outperforms adaptive neuro-fuzzy inference system techniques. Wang and Hu^68^ found that LS-SVM is recommended over SVM and LSSVM for large-scale problems because its solution technique is more efficient.

To minimize the model output (Eq. (3)), the Lagrange method using Eqs. (4)–(6) was adopted in this study.345
where *w* is the weight vector, *g* ϵ* R*^+^ is the penalty parameter, *ḉ*_*i*_ is an error deviation, and ƥ (.) is mapping to a high-dimensional space.6

The kernel function K is computed using Eq. (7), which is substituted into Eq. (8) for the linear transformation of Eq. (6) as proposed by Karush–Kuhn–Tucker (KKT) conditions.7

8 $$ \left( {\begin{array}{*{20}c} 0 & {1_{l}^{T} } \\ {1_{l} } & {K + g^{ - 1} 1_{l} } \\ \end{array} } \right)\left( {\begin{array}{*{20}c} b \\ \alpha \\ \end{array} } \right) = \left( {\begin{array}{*{20}c} 0 \\ y \\ \end{array} } \right) $$

where $y = \left[ {y_{1} ,y_{2} ,. \ldots y_{l} } \right]^{T}$, $l_{i} = \left[ {1, \ldots \ldots ..l} \right]^{T}$, $l_{i}$ is $l_{i} h$ ordered unit matrix, $\alpha = \left[ {\alpha_{1} ,. \ldots \alpha_{l} } \right]^{T}$ K is the performance and generalization ability noted by Wang et al.^66^. In this study, Python-based LSSVM was utilized for training using seven inputs to predict the PPV. A large kernel width control factor value was employed during modeling training to optimize the model learning process and avoid overfitting, as proposed by Félix et al.^69^. However, the function-based LSSVM models are employed, having a matrix dimension of 7:1 (input: output). Each model has been configured with a gamma of 3 and a sigma of 10.

#### Support vector machine (SVM)

According to Reddy, the Support Vector Machine is the most extensively used machine learning module in geotechnical engineering and tunnelling^70^. Also, the Support vector regression (SVR) learning prediction procedure is based on statistical learning theory. This supervised learning algorithm has various characteristics, and it avoids over-fitting due to simple decision limits^71^. Kurani et al.^72^ used SVM to forecast the stock market. SVM reduces overfitting by employing kernel functions and regularization settings^73^. Li and Mei^74^ have predicted the tunnel crown displacement induced by blasting excavation using hybrid SVR models. Also, Li et al.^75^ have employed hybrid SVR models to assess the fly-rock distance in surface mining. Support Vector Regression (SVR), or regression-based SVM in the literature, can be described as a linear SVR using the formula in Eq. (9).

9 $$ f\left( x \right) = \sum\limits_{i}^{n} {\varphi \left( {x_{i} } \right)\,} w + b $$

**Figure 6:**
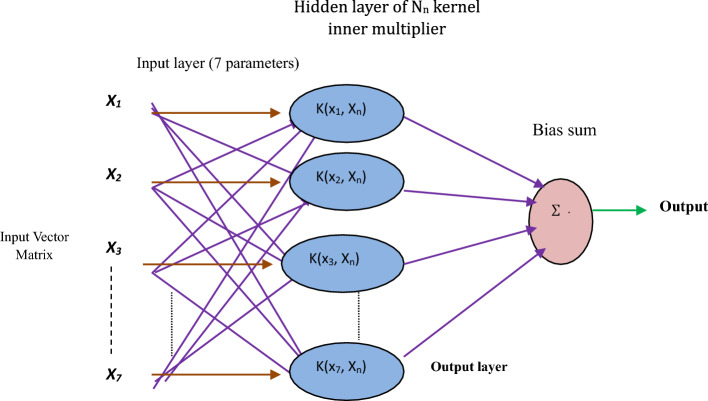
Support vector machine structure.

In which *f*(*x*) indicates the model output, *x*_*i*_ signifies an input variable, *φ* denotes nonlinear mapping, and *w* and *b* are the regression function's weight vector and bias. Figure 6 depicts the structure of the support vector machine used in this study, which includes an input layer, a hidden layer, and an output layer.

The support vector machine model is configured with default hyperparameters, i.e., kernel function (= Gaussian), box constraint (= 0.0149), kernel scale (= 0.1566), epsilon (= 0.0689), sequential minimal optimization solver, gap tolerance (= 1.0E − 0.3), and bias (= 15.0950). So, the best prediction can be achieved by the SVM model. Furthermore, the SVM model is optimized by the Bayesian regularization algorithm. The iterations, maximum training time, and number of grid divisions are kept default, i.e., 30, 300 s, and 10, respectively.

#### Gaussian process regression (GPR)

The Gaussian process represents an output parameter by collecting random variables, any finite number of which have a joint Gaussian distribution, as explained by^76^. GPR applications have been observed for decades. Keprate et al.^77^ used GPR to forecast stress intensity factors in a bit to analyze fatigue degradation in the offshore pipe. Arthur et al.^78^ used GPR to reduce blast impact in a Ghana open pit mine. The analysis found that the GPR suggested model was more accurate than the US Bureau of Mines model, the Langefors and Kihlstrom model, the Ambraseys-Hendron model, and the Indian Standard model. GPR model output Y(X) for N dataset using Eq. (10) with the Gaussian regression model present in Eq. (11). Where (*x*) maps an input vector *x* into a feature space.

10 $$ {\text{F}}\left( {\text{x}} \right) = p\left( {\text{x}} \right)^{{\text{T}}} {\text{w}} $$

11 $$ y/X,K \sim N\left( {0, X, X} \right) + \sigma_{n}^{2} {\rm I}) $$

where *K*(*X*, *X* ) is the covariance matrix, and *k* is the covariance function or kernel function. For some test inputs X*, the joint distribution of the target and function values under the prior is calculated using Eq. (12).

12 $$ \left[ {\begin{array}{*{20}c} y \\ {f*} \\ \end{array} } \right] = N\left( {O,\begin{array}{*{20}c} {K\left( {X,X} \right) + \sigma_{n}^{2} {\text{\rm I}})} & {K\left( {X,X*} \right)} \\ {K\left( {X*,X} \right)} & {K(K*,X*} \\ \end{array} } \right) $$

The squared exponential covariance functions used in this study are parameterized in terms of hyper-parameters as shown in Eq. (13). The kernel function of the Gaussian process model is a positive semi-definite, such as linear kernel function, polynomial kernel function, Gaussian kernel function, and recurrent kernel function. The log-likelihood of training data is transformed using Eq. (14).

13 $$ k\left( {xp,xq} \right) = \sigma_{n}^{2} {\text{{\rm I}exp}}( - \frac{1}{{2{\text{l}}^{2} }}(xp - xq)^{2} + \sigma_{n}^{2} \delta_{pq} $$

14 $$ l\left( \theta \right) = \left( {\left( { - \frac{1}{2}y^{T} \left( {k + \sigma_{n}^{2} I} \right)} \right)^{ - 1} \times y} \right) - \frac{1}{2}log/k + \sigma_{n}^{2} I/ - \frac{n}{2}log2\pi $$

Where ɵ is the vector containing all the hyperparameters as expressed in Eq. (15).

15 $$ \theta = \left\{ {l,\sigma_{f}^{2} ,\sigma_{n}^{2} } \right\} $$

The output result is optimized by maximizing the log-likelihood of the training data using conjugate descent as a numerical optimization method. Default hyperparameters configure the Gaussian process regression model to obtain the best ground vibration prediction in this study. The basic function (zero, constant, and linear), kernel function (= exponential), kernel parameters (= 0.7183; 3.0527), sigma (= 0.3313), iterations (= 30), maximum training time (= 300), and number of grid divisions (= 10) are selected as default. The GPR model is also optimized by the Bayesian optimization algorithm.

#### Decision tree (DT) and ensemble tree (ET)

According to Khan et al.^79^, the machine learning (ML) algorithm is an essential component of the intelligent algorithm. Ensemble modeling is a process in which numerous models are developed to predict an outcome, either by employing various modeling techniques or a variety of training data sets. The ensemble model then aggregates the predictions of each base model, yielding a single final prediction for the unknown data. Yin et al.^80^ found that ensemble models that used KNN and RNN fared the best across all ensemble models. Although these ensemble models have a high level of prediction accuracy, they have several practical issues that prohibit them from being extensively deployed^81^. Overfitting is a common problem with ensemble models. Various challenges necessitate different combined methods, and there should be a distinction between the basis classifiers and the combination technique, and the base classifier must be chosen. Otherwise, performance will not be able to improve^82^. Many hyperparameters were modified to produce an optimum result when creating the proposed PPV model utilizing the ensemble model. The ensemble model's parameter settings significantly impact capacity^81^. Because decision tree techniques closely mirror human reasoning and are simple to understand, they have been frequently employed to develop classification models. The modeling technique is also known as a regression tree because it involves a calculation process that resembles a tree structure. Dauji^83^ states that DT flows from a root node (decision box) to decision boxes based on the 'yes' or 'no' decision output. As a result, during the modeling step, the model space is partitioned into subspaces, each with its own decision rule. The suggested Techniques for avoiding too many domain splits neighboring models were applied while creating the proposed DT model for PPV, as detailed in Maimon and Rokach^84^ and Jekabsons^85^ studies. The PPV prediction input data hyper-space was partitioned to obtain the desired maximum decrease in standard deviation in the DT M5 algorithm for PPV estimation. The decision (split criterion = MSE, min-parent = 12, min leaf = 6, max splits = 129, tolerance = 1.0E-6) and ensemble (N-learn = 497, method = bag) tree models are configured for better prediction with 1 to 10 leaves and a Bayesian optimization algorithm.

#### Artificial neural networks (ANN)

The artificial neural network (ANN) is a computational modeling technique that mimics the structure and functionality of natural neural networks. This artificial intelligence modeling method is inspired by how the organic brain system functions^50,86^. ANN is a three-layer processing architecture that includes an input layer, a hidden layer, and an output layer collaborating to solve specific issues. These networks fit nonlinear and perceiving patterns^87^. Fissha et al.^88^ introduced the application of Bayesian-based ANN to improve the Mikurahana quarry blasting impact, demonstrating a new advantage of neural networks for ground improvement. Bhatawdekar et al.^89^ developed a soft computing model for estimating fly rock distance using different input variables, such as hole diameter, burden, stemming length, rock density, charge-per-meter, powder factor (PF), blast-ability index (BI), and weathering index datasets using hybrid ANN approaches. This study used an ANN technique with Bayesian Regularization algorithms to construct a prediction model for PPV. One hundred sixty-two datasets were used for the proposed model construction, with 80% training and 20% testing databases. Khatti and Grover^90^ analyzed the impact of hidden layers and neurons on the ANN model's performance in predicting compacted soil's compaction parameters. In the published work, authors observed that the performance of the ANN model increases with neurons, i.e., up to 10. On the other side, the overfitting occurs due to several hidden layers. Therefore, it has been decided to configure the ANN model with eight neurons and one hidden layer. Hence, the ANN model has been structured as 7-8-1 (where seven variables at the input layer, and eight neurons connect the input layer with one hidden layer), and the sigmoid transfer function scored best for PPV prediction. An excellent configuration of neural networks can achieve the best ground vibration prediction. This study configures the neural network model with 1000 epochs, eight hidden layers, a sigmoid activation function, 0.000346 gradient, 0.01 learning rate, and a Bayesian regularization algorithm.

#### Long short-term memory (LSTM)

**Figure 7:**
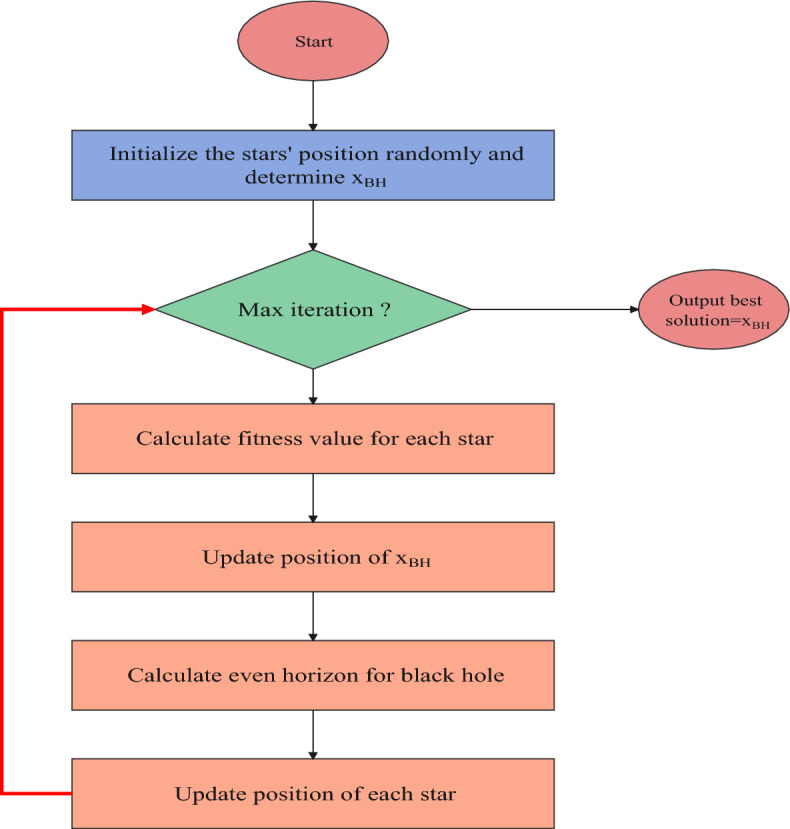
Flow chart of black hole optimized LSTM model.

According to Yu et al., LSTM is a variety of recurrent neural networks (RNNs) capable of learning long-term dependencies, especially in sequence prediction problems. Mehedi et al.^91^ explained that LSTM contains a memory cell that stores performance historical data. This memory cell employs three regulating gates, called the input, forget, and output gates, which allow the LSTM network to add or delete content from the cell state. The cell state ${C}_{t}$ of a typical LSTM includes both sigmoidal layer function *σ* and a point-wise multiplication operator incorporated to control the network information through the three processing gates (forget, input, and output gates). The input gate expression is presented in Eq. (16) for decision-making regarding the position of $x_{t}$, and the storage decision in the cell state.

16 $$ i_{t} = { }\sigma \left( {W_{i} h_{t - 1} + W_{i} h_{t} + b_{i} } \right) $$

where $i_{t}$ is the input gate, $W_{i}$ and $b_{i}$ represents the input weight and bias of the input gate, respectively. After each state, new data $x_{t}$ is sent to the network at each time instant t; the forget gate response at every instant is represented by Eq. (17).

17 $$ f_{t} = { }\sigma \left( {W_{f} h_{t - 1} + W_{f} h_{t} + b_{f} } \right) $$

where $W_{f}$ and $W_{f}$ represents the input weight and bias of the forget gate, respectively, $f_{t}$ denotes the forget gate, $h_{t - 1}$ represent the output block memory. A new vector $\widetilde{{C_{t} }}$ is generated using tanh layer function as expressed in Eq. (18) to introduce the cell state $C_{t}$ process stage condition.

18 $$ \widetilde{{C_{t} }} = { }tanh\left( {W_{c} h_{t - 1} * W_{c} x_{t} *b_{c} } \right) $$

where $W_{c}$ is the cell state weight and $b_{c}$ is the cell state bias, and * represents the Hadamard product^92^. The model output memory block $h_{t}$ is created by using the output gate $o_{t}$, and another tanh layer, as expressed in Eqs. (19) and (20).

19 $$ o_{t} = { }\sigma \left( {W_{o} h_{t - 1} + W_{o} x_{t} + b_{o} } \right) $$

20 $$ h_{t} = o_{t} *{\text{tanh}}\left( {c_{t} } \right) $$

where $$h_{t} $$ and $$h_{t}$$ indicates the input weight and bias of the output gate. In this work, a new LSTM intelligent optimizer based on the blackhole optimization algorithm was applied for variable position optimization. The BH algorithm is an example of a population-based approach with some characteristics in common with other such approaches. Like previous population-based algorithms, this one generates and randomly distributes a population of candidate solutions to the problem across the search space. Figure 7 presents the black hole algorithm flow chart.

A black hole (BH) is a region of space with a very high mass-to-volume ratio. Particle mass is concentrated in the singularity, also known as the core of the BH. The event horizon is a region of extremely strong gravitational pull that surrounds the black hole singularity and prevents nearby particles from escaping. Each possible answer is called a “star” in the BHA; therefore, we begin by randomly building stars in the search spaces. This is done to find the best possible answer. Then, it examines each star to get the best possible answer or BH. As the stars keep moving in the same direction as the BH, it gradually absorbs them. The mechanism by which the stars move is described by Eq. (21). The BH algorithm is an example of a population-based approach with some characteristics in common with other such approaches. Like previous population-based algorithms, this one generates and randomly distributes a population of candidate solutions to the problem across the search space.

21 $$ x^{i} \left( {t + 1} \right) = x^{i} \left( t \right) + r\left[ {x_{BH} - x^{i} \left( t \right)} \right] $$

In which *x*^*i*^(*t*) and *x*^*i*^(*t* + 1) indicate the locations of the *i*th star at repetitions *t* and *t* + 1, respectively. In addition, *r* signifies a random value between (0, 1), and x_BH_ is the BH's location in searching spaces. The LSTM models are configured with epochs to 1000, batch size of 52, gradient threshold of 0.022, initial learning rate of 0.00017, learn rate drop period of 191, and learn rate drop factor of 0.1. The alpha and beta of the blackhole optimization algorithm have been selected as 0.4 and 0.9, respectively, for the blackhole optimized LSTM model.

### Performance evaluation

Several metrics are available in statistics, which is used in computational mechanics. Sixteen performance metrics have been implemented in this research. The mathematical formulation of the performance metrics is as follows^90,93–97^:

Root Mean Square Error (RMSE)

22 $$ RMSE = \sqrt {\frac{1}{n}\mathop \sum \limits_{i = 1}^{n} \left( {\alpha - \beta } \right)^{2} } $$

Mean Absolute Error (MAE)

23 $$ MAE = \frac{1}{n}\mathop \sum \limits_{i = 1}^{n} \left| {\left( {\omega - \alpha } \right)} \right| $$

Coefficient of determination (R^2^)

24 $$ R^{2} = \frac{{\mathop \sum \nolimits_{i = 1}^{r} \left( {\alpha - \beta } \right)^{2} - \mathop \sum \nolimits_{i = 1}^{r} \left( {\alpha - \omega } \right)^{2} }}{{\mathop \sum \nolimits_{i = 1}^{r} \left( {\alpha - \beta } \right)^{2} }} $$

Coefficient of correlation (r)

25 $$ r = \frac{{\sum \left( {\alpha_{i} - \overline{\beta }} \right)\left( {\omega_{i} - \overline{\omega }} \right)}}{{\sqrt {\sum \left( {\alpha_{i} - \overline{\beta }} \right)^{2} \sum \left( {\omega_{i} - \overline{\omega }} \right)^{2} } }} $$

Mean Absolute Percentage Error (MAPE)

26 $$ MAPE = \frac{1}{n}\mathop \sum \limits_{i = 1}^{n} \left| {\frac{\alpha - \omega }{\alpha }} \right|*100 $$

Weighted Mean Absolute Percentage Error (WMAPE)

27 $$ WMAPE = \frac{{\mathop \sum \nolimits_{i = 1}^{n} \left| {\frac{\alpha - \omega }{\alpha }} \right|*\alpha }}{{\mathop \sum \nolimits_{i = 1}^{n} \alpha }} $$

Variance Accounted For (VAF)

28 $$ VAF = \left( {1 - \frac{{var\left( {\alpha - \omega } \right)}}{var\left( \alpha \right)}} \right)*100 $$

Root mean square error to observation's standard deviation ratio (RSR)

29 $$ RSR = \frac{RMSE}{{\sqrt{\frac{1}{N}}  \mathop \sum \nolimits_{i = 1}^{N} \left( {{\upalpha } - {\upbeta }} \right)^{2} }} $$

Nash–Sutcliffe efficiency (NS)

30 $$ NS = 1 - \frac{{\mathop \sum \nolimits_{i = 1}^{n} \left( {{\upalpha } - \omega } \right)^{2} }}{{\mathop \sum \nolimits_{i = 1}^{n} \left( {{\upalpha } - {\upbeta }} \right)^{2} }} $$

Legate and McCabe's index (LMI)

31 $$ LMI = 1 - \left[ {\frac{{\mathop \sum \nolimits_{i = 1}^{n} \left| {\alpha - \omega } \right|}}{{\mathop \sum \nolimits_{i = 1}^{n} \left| {\alpha - {\upbeta }} \right|}}} \right] $$

Normalized Mean Bias Error (NMBE)

32 $$ NMBE = \frac{{\frac{1}{N}\mathop \sum \nolimits_{i = 1}^{n} \left( {\omega - {\alpha }} \right)^{2} }}{{\frac{1}{N}\mathop \sum \nolimits_{i = 1}^{n} {\upalpha }}} $$

a-20 index

33 $$ a20 index = \frac{m20}{H} $$

Index of Agreement (IOA)

34 $$ IOA = 1 - \frac{{\mathop \sum \nolimits_{i = 1}^{n} \left( {\omega - \alpha } \right)}}{{2\mathop \sum \nolimits_{i = 1}^{n} \left( {\alpha - \beta } \right)}} $$

Index of Scatter (IOS)

35 $$ IOS = \frac{RMSE}{{Avg. of Actual Values}} $$

Performance Index (PI)

36 $$ PI = R^{2} + \left( {VAF/100} \right) - RMSE $$

Bias Factor (BF)

37 $$ BF = \frac{1}{N}\mathop \sum \limits_{i = 1}^{n} \frac{\alpha }{\omega } $$

where β is the mean of the real values, $k$ is the number of independent variables, n presents the total number of data, $\overline{\omega }$ is the mean of the predicted value, α and $\omega$ are the real and predicted i^th^ values, m20 is the ratio of experimental to the predicted value (0.8 to 1.2), and H is the total number of data samples. The value of R more than 0.8 (R^2^ = 0.64) shows strong, between 0.2 (R^2^ = 0.4) to 0.8 (R^2^ = 0.64) presents good, and less than 0.2 (R^2^ = 0.4) presents weak correlation between pairs of data^98^. A perfect predictive model always has a performance equal to the ideal value mentioned in Table 3.

**Table 3:** Ideal value of the different performance indicators.

Indicators	Value	Indicators	Value
RMSE	0	NMBE	0
MAE	0	NS	1
R^2^	1	LMI	0
PI	2	RSR	0
MAPE	0–100	a20-index	100
WMAPE	0	IOA	1
VAF	100	IOS	0
R	1	BF	1

### Nonlinear sensitivity analysis

**Figure 8:**
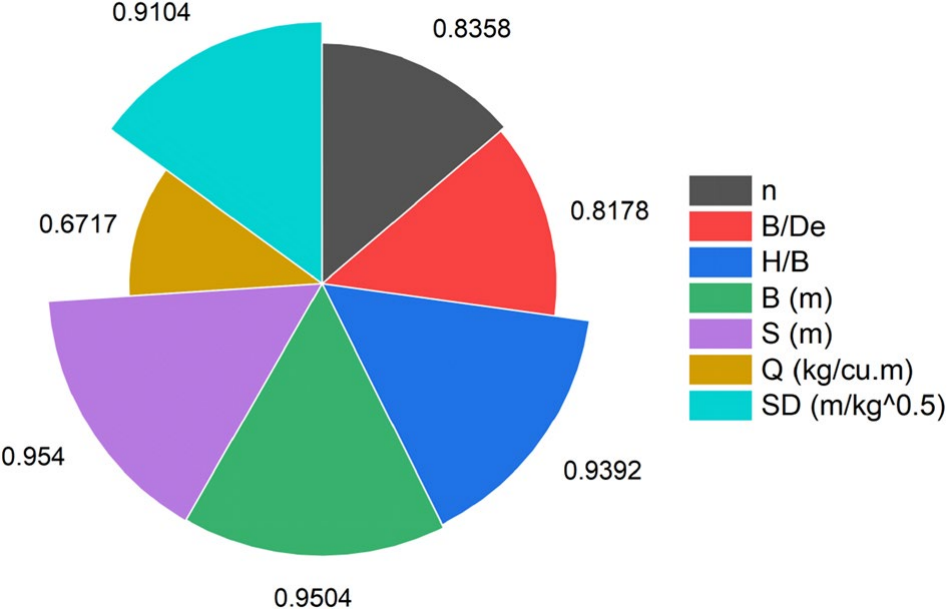
Illustrations of sensitivity analysis for PPV (ground vibration).

Sensitivity analysis is used to identify the variables that will affect the prediction the most. Sensitivity analysis comes in both global and local forms. The sensitivity analysis can be carried out using several techniques, and cosine amplitude is one of them. The cosine amplitude technique has been implemented in this study. The mathematical expression of the cosine amplitude method is^93^:

38 $$ CAM = \frac{{\mathop \sum \nolimits_{c = 1}^{n} \left( {X_{ic} *X_{jk} } \right)}}{{\sqrt {\mathop \sum \nolimits_{c = 1}^{n} X_{ic}^{2} } \mathop \sum \nolimits_{c = 1}^{n} X_{jk}^{2} }} $$

where $X_{ic}$ is input parameters n, B/De, H/B, B, S, Q, and SD, and $X_{jk}$ is output parameter PPV (ground vibration). A strong influencing input variable always has a CAM value near one. In this study, 162 data points have been collected from the field. The sensitivity analysis result has been drawn, as depicted in Fig. 8.

Figure 8 illustrates that the input parameters, such as n, B/De, H/B, B, S, and SD, highly influence the ground vibration prediction during blasting. Comparing all input variables, the Q variable (= 0.6717) influences the PPV (ground vibration) prediction less than other input variables.

## Results and discussion

### Results simulation

In this work, PPV1, PPV2, PPV3, PPV4, PPV5, PPV6, PPV7, PPV8, PPV9, PPV10, and PPV11 models based on MLR, SVM, GPR, DT, ET, L-LSSVM, P-LSSVM, G-LSSVM, ANN-BR, LSTM, and LSTM-BA approaches have been employed, performed, and analyzed. The fifteen metrics have measured the training (TR) and testing (TS) performances of all models, as summarized in Table D (refer Appendix). Table D demonstrates that the GPR model PPV3 has higher performance (TS = 0.8832, TR = 0.9999) than other conventional models, i.e., MLR, SVM, DT, and ET. The model PPV3 performs better if the database is limited/ small^99^. It has been measured that model PPV3 predicts the ground vibration with RMSE = 0.0737 mm/s, MAE = 0.0580 mm/s, MAPE = 12.2871 mm/s, WMAPE = 0.1135 mm/s, and NMBE = 0.0106, in the TS phase. Comparing LSSVM models, i.e., PPV6 (linear kernel-based), PPV7 (polynomial kernel-based), and PPV8 (Gaussian kernel-based), demonstrates that LSSVM model PPV8 has outperformed models PPV6 and PPV7, with the higher TS performance (RMSE = 0.0487 mm/s, MAE = 0.0382 mm/s, R = 0.9751, MAPE = 10.3971 mm/s, VAF = 95.07, WMAPE = 0.0749 mm/s, NS = 0.9038, PI = 1.8528, BF = 0.9054, NMBE = 0.0047 mm/s, LMI = 0.3214, RSR = 0.3102, a20-index = 96.88, IOA = 0.8393, and IOS = 0.0955).

**Figure 9:**
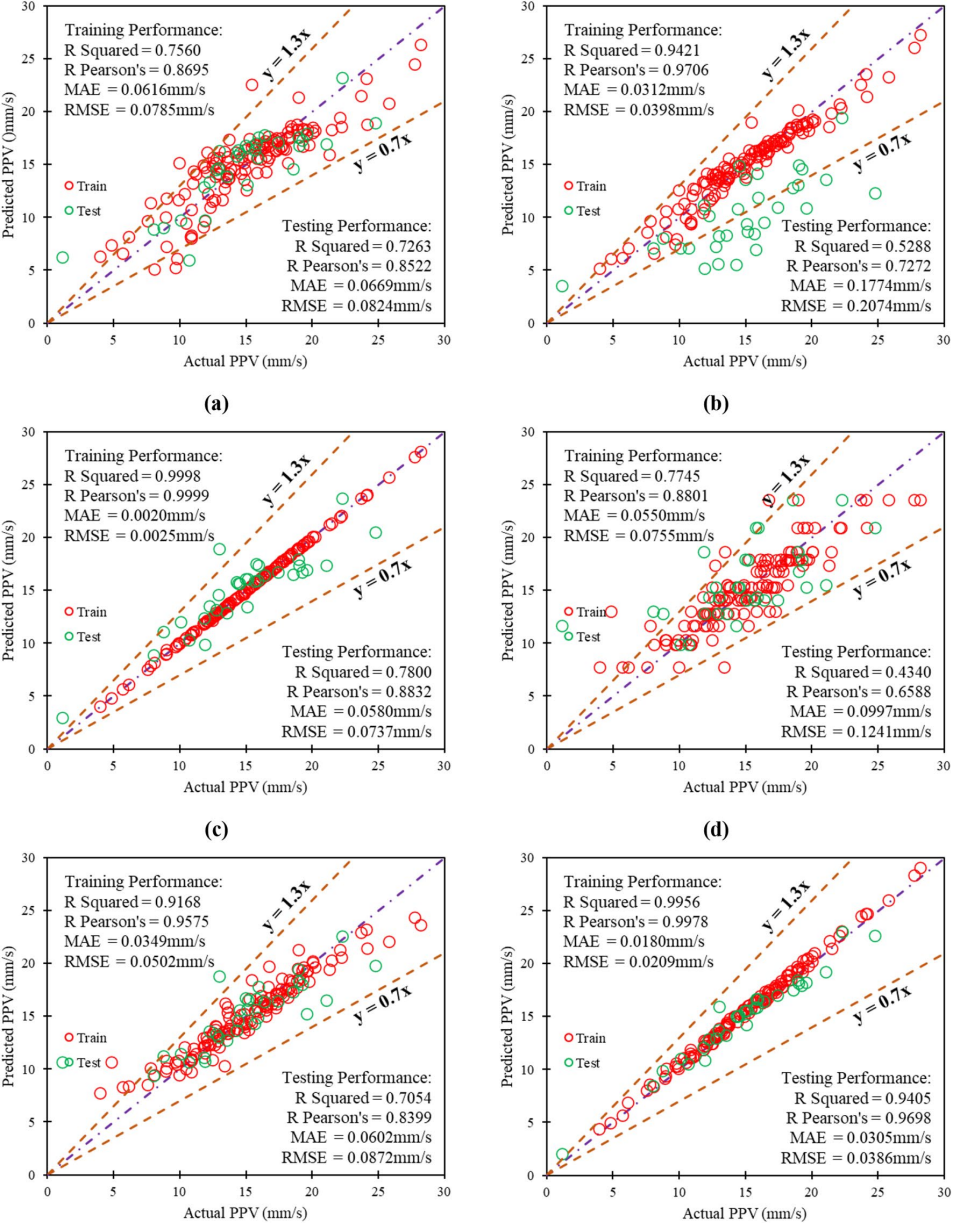

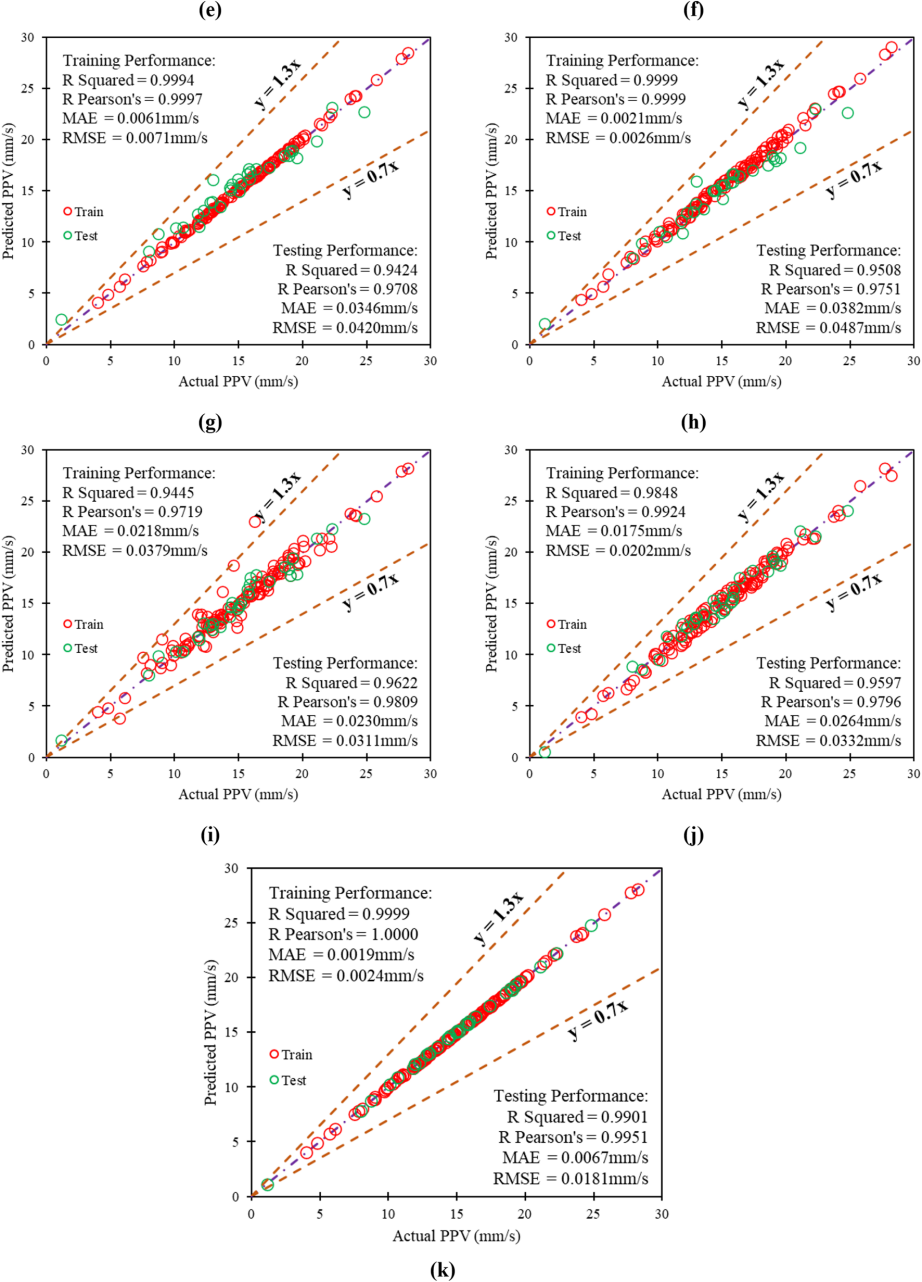
Illustration of the relationship between actual and predicted PPV using models: (**a**) PPV1, (**b**) PPV2, (**c**) PPV3, (**d**) PPV4, (**e**) PPV5, (**f**) PPV6, (**g**) PPV7, (**h**) PPV8, (**i**) PPV9, (**j**) PPV10, and (**k**) PPV11.

Moreover, the deep learning approach based-models, i.e., ANN-BR (model PPV9), LSTM (model PPV10), and LSTM-BA (model PPV11), have been compared. The performance comparison of models PPV9 and PPV10 demonstrates that the ANN-BR model has attained higher TR (R = 0.9719) and TS (R = 0.9809) performance in predicting ground vibrations during blasting. The comparison shows that model PPV9 predicts ground vibration with the least residuals in the TR and TS phases. In addition, the blackhole algorithm optimized model PPV10 has gained over 99% (TR = 1.0000, TS = 0.9951) accuracy in both phases. Also, model PPV11 has estimated the ground vibration with the least prediction error in the testing phase (RMSE = 0.0181 mm/s, MAE = 0.0067 mm/s, MAPE = 3.8927 mm/s, WMAPE = 0.0131 mm/s, and NMBE = 0.0006), comparatively higher than other models. The overall comparison reveals that PPV11 is the optimum performance model for assessing peak particle velocity (ground vibration) during blasting. A statistical relationship is drawn between actual and predicted PPV using models PPV1 to PPV11, as depicted in Fig. 9.

### Visual interpretation of results

The visual interpretation of results has been drawn for a better understanding and analysis. The score analysis has been discussed in this section, and the results are presented. The second section plots the regression error characteristics curves for the training and testing phase.

#### Score analysis

**Figure 10:**
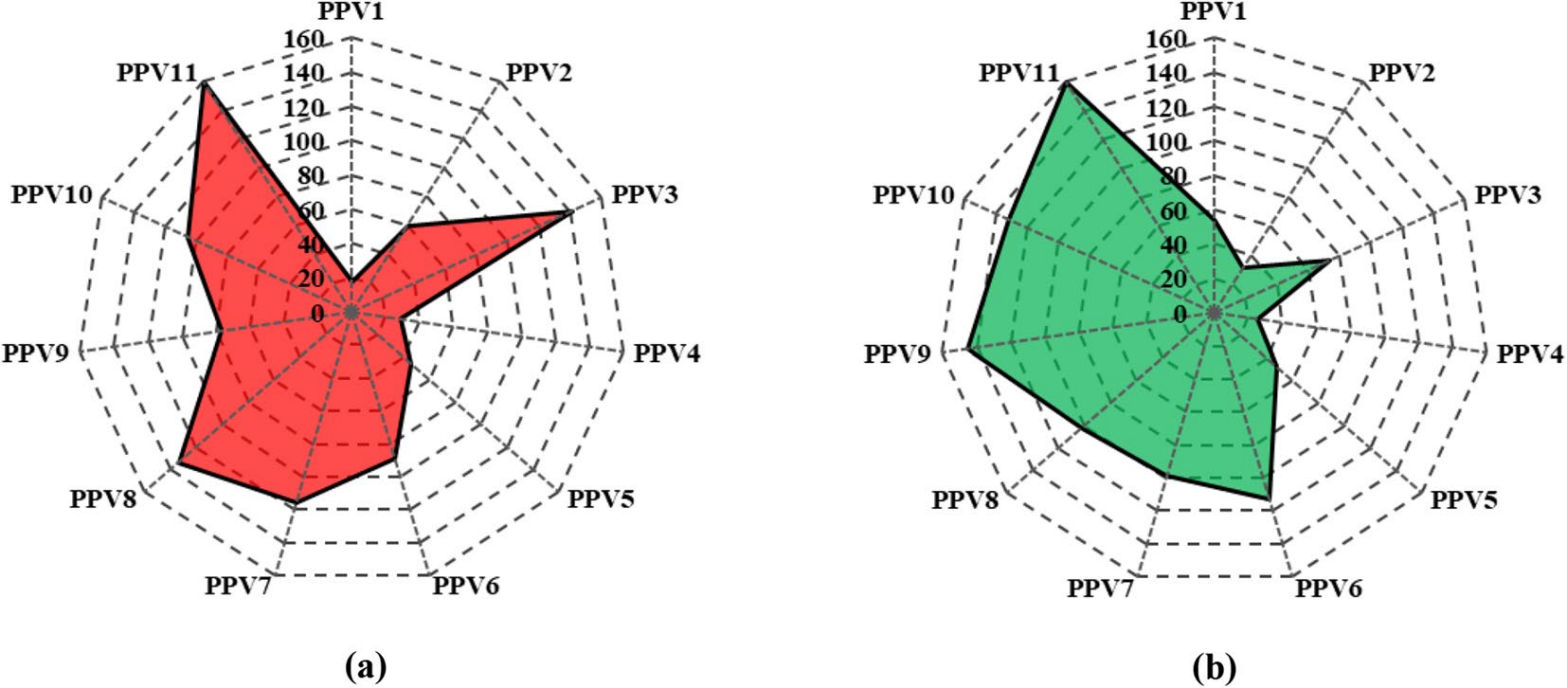
Depiction of score analysis results for computational models in (**a**) TR and (**b**) TS phase.

The score analysis compares the effectiveness of computational models through statistical analysis. A score of n is assigned to the model for selecting the best value for each performance indicator (in this study, n = 11; see soft computing models taken into account in the analysis). The higher and lower values of performance indicators in score analysis demonstrate the better and worse training and testing examples for the models. The final model score is calculated by averaging the performance indicator scores from each training and testing phase. The training and testing scores are then added to determine a model's total score. The score analysis results for training and testing performances of the computational models PPV1 to PPV11 are summarized and depicted in Table E and graphically presented in Fig. 10a and b.

Figure 10a and b and Table 6 illustrate that model PPV11 has obtained higher scores in training and testing. Hence, the LSTM-BA model PPV11 is the optimum performance model for assessing ground vibration (PPV) during blasting. It has also been observed that the decision tree model PPV4 has obtained the lowest score and is identified as the poor-performance soft computing model in the present research.

#### Regression error characteristics (REC) curve

**Figure 11:**
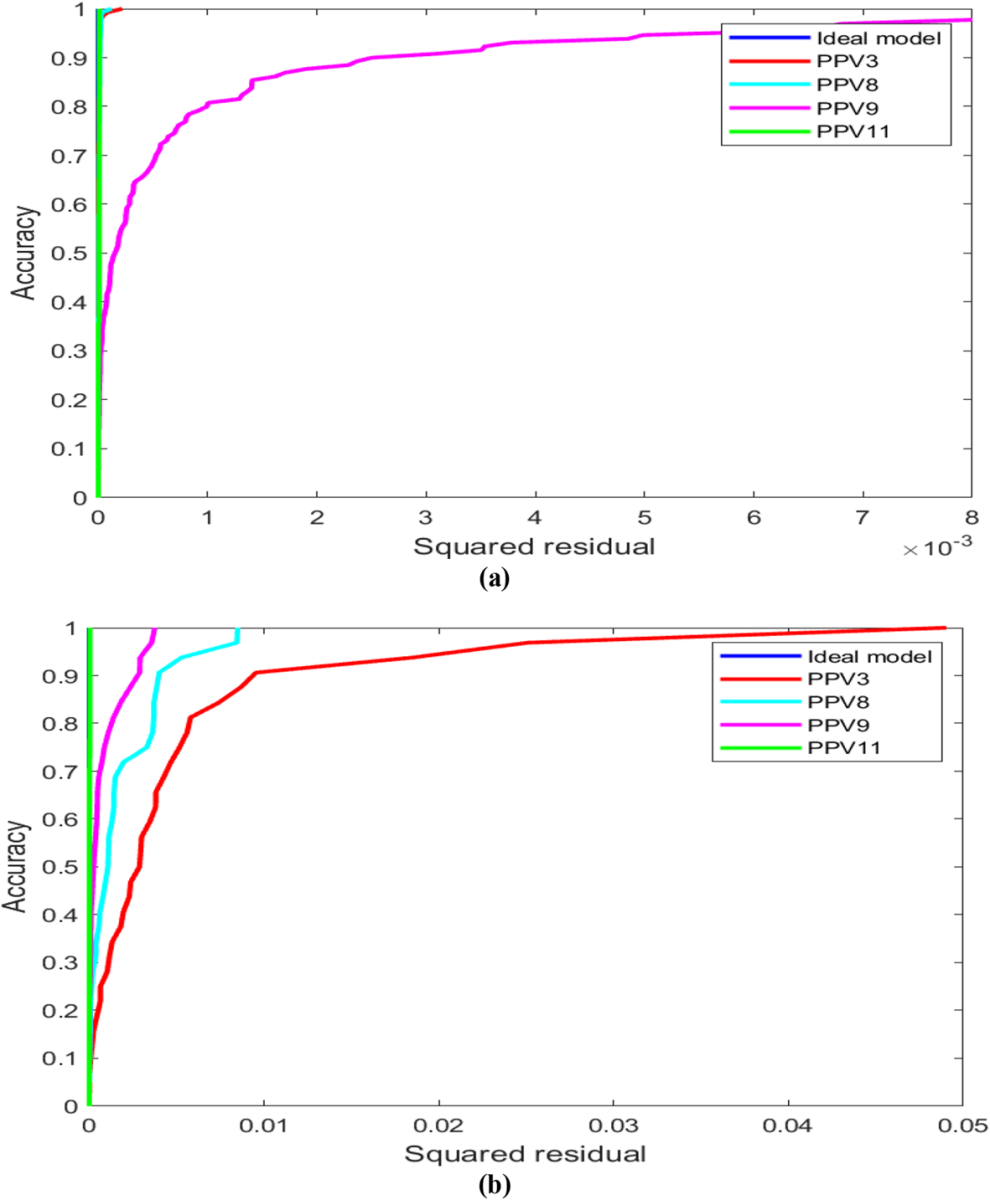
Depiction of REC curve for soft computing models in (**a**) training and (**b**) testing phase.

Regression Error Characteristic (REC) curves generalize ROC curves to regression. The percentage of predicted points that fall inside the tolerance is shown on the y-axis of REC curves, and the error tolerance is on the x-axis. The resulting curve provides an estimation of the error's cumulative distribution function. In the present research, REC curves have been plotted for models PPV3 (machine learning model), PPV8 (advanced machine learning model), PPV9 (deep learning model), and PPV11 (hybrid learning model) and compared with each other to find the optimum performance model, as shown in Fig. 11, along with the AOC values, mentioned in Table 4.

**Table 4:** AOC values for soft computing models.

Phase	Actual	PPV3	PPV8	PPV9	PPV11
Train	0.00E+00	5.70E−06	6.45E−06	1.20E−03	**5.43E−06**
Test	0.00E+00	4.78E−03	1.72E−03	7.21E-04	**1.48E−05**

*Bold values present to the optimum performance model.

Table 4 shows that model PPV11 has attained the least AOC, i.e., 5.43E−06 in training and 1.48E−05 in the testing phase, close to the AOC of actual ground vibration. Hence, model PPV11 is an optimum performance model.

### Anderson darling test

**Figure 12:**
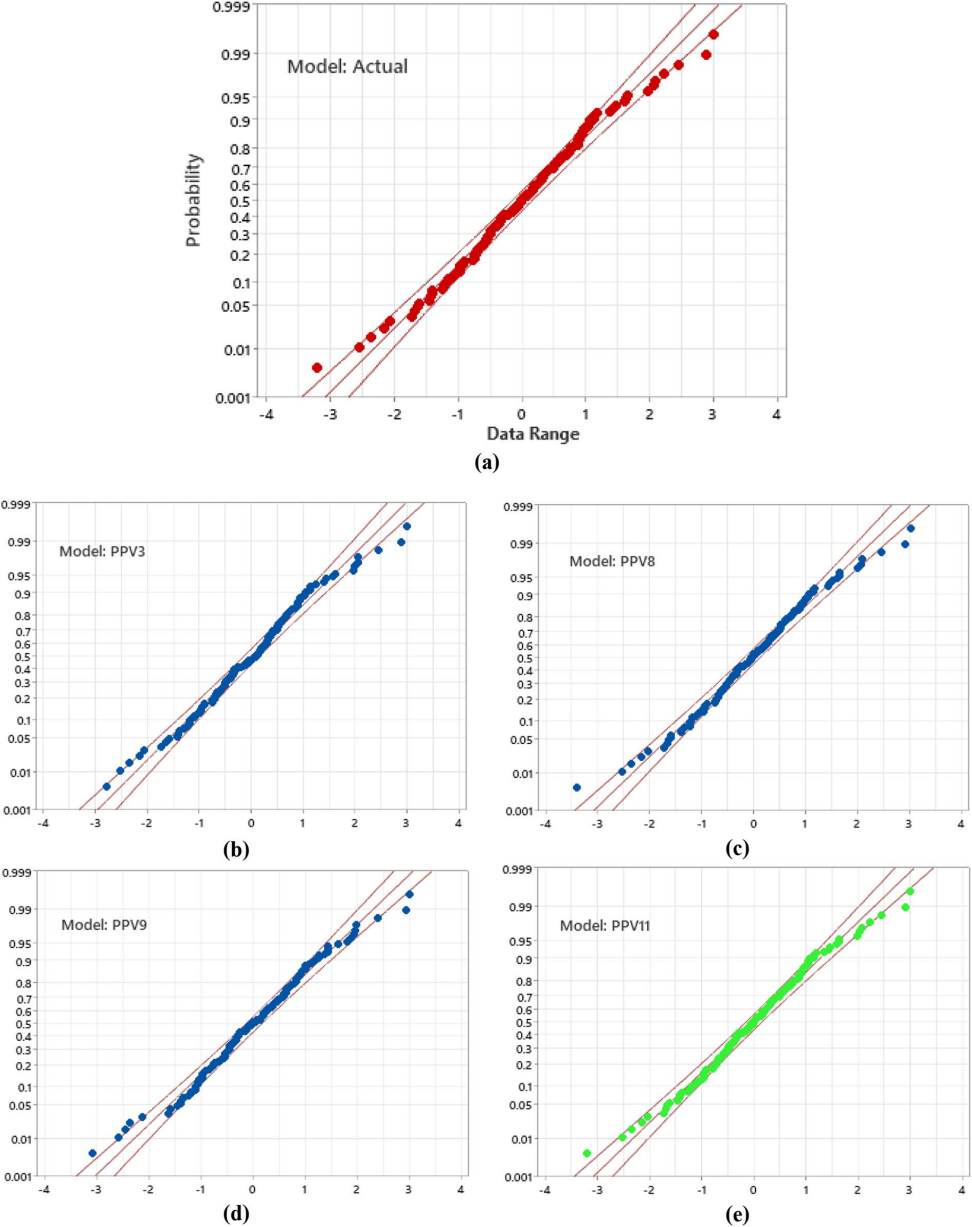
Illustration of AD test for model (**a**) actual, (**b**) PPV3, (**c**) PPV8, (**d**) PPV9, and PPV11.

The Anderson–Darling test is utilized to test if an example of information came from a populace with a particular dissemination. It is a change of the Kolmogorov–Smirnov (K-S) test and gives more weight to the tails than the K-S test. The K-S test is without circulation as the basic qualities don't rely upon the particular dissemination being tried (note that this is valid just for a completely determined conveyance; for example, the boundaries are known). The Anderson–Darling test utilizes the particular conveyance in ascertaining basic qualities. In the present research, the Anderson–Darling (AD) test has been performed for models PPV3, PPV8, PPV9, and PPV11. The results obtained from the AD test are graphically presented in Fig. 12, along with Table 5.

**Table 5:** Anderson–Darling test results for the whole database.

Model ID	Database	AD Value	*p*-value
Actual	162	0.443	0.0283
PPV3		0.628	
PPV8		0.504	
PPV9		0.456	
**PPV11**		**0.438**	

*Bold values present to the optimum performance model.

The AD test has been performed for the entire database (training + testing). Table 5 demonstrates that the actual model has a 0.0283 p-value, less than the significance level (0.05), showing the rejection of the null hypothesis of normality. The AD value of model PPV11 is closest to the actual value, which confirms the superiority of the LSTM-BA model PPV11 over the other machine, advanced machine, and deep learning models.

### Results analysis

**Figure 13:**
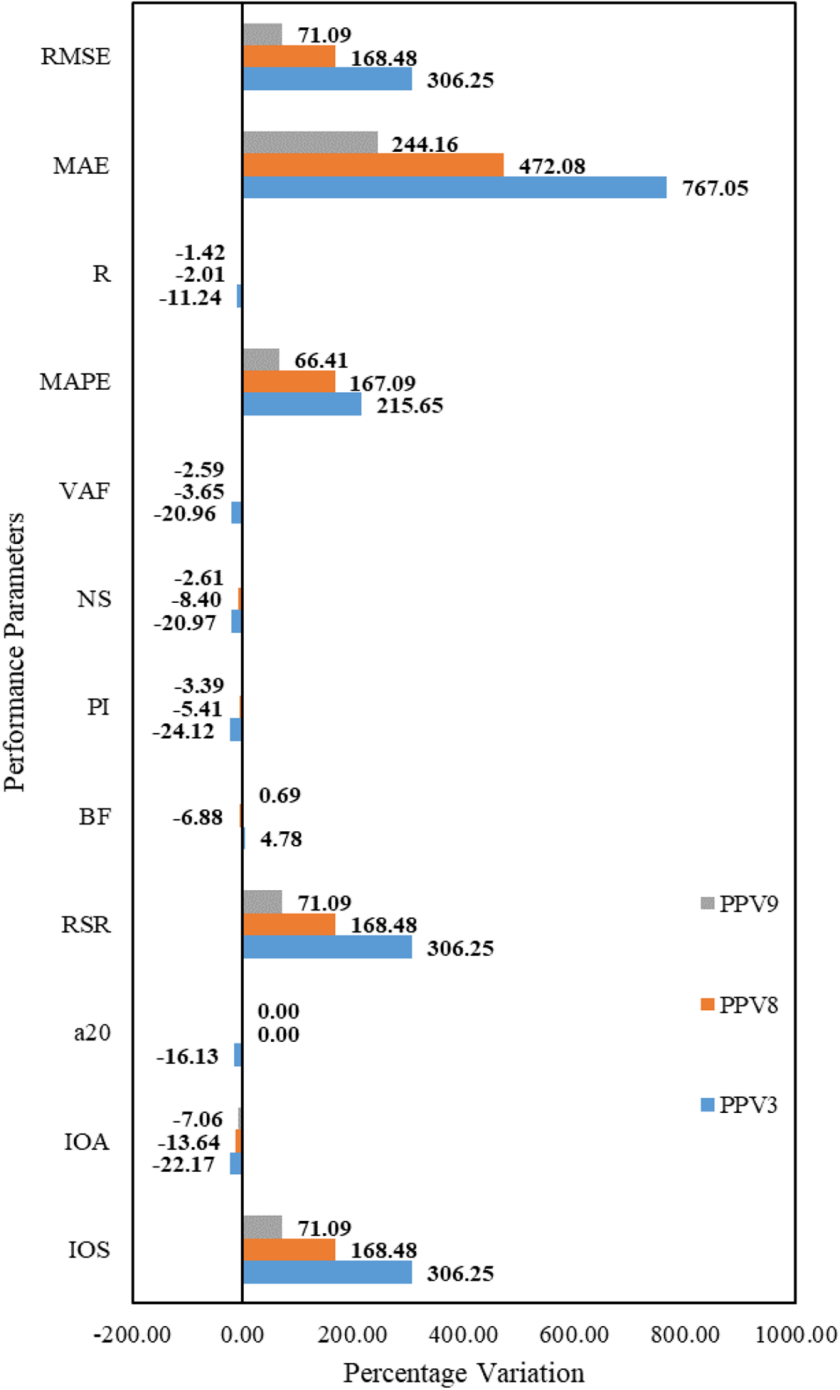
Illustration of performance variation for models PPV3, PPV8, and PPV8.

In this work, eleven computational models have been developed, performed, and analyzed in predicting ground vibration during blasting. The analysis of fifteen metrics reveals that the GPR model PPV3, Gaussian kernel function-based LSSVM model PPV8, ANN-BR model PPV9, and blackhole optimized-LSTM model PPV11 have performed better. Figure 13 illustrates the test performance variation for models PPV3, PPV8, and PPV9 wrt PPV11. Figure 13 shows that model PPV3 has attained 306.25% higher RMSE, RSR, and IOS than models PPV8 and PPV9. Also, model PPV3 has gained VAF = 20.96%, NS = 20.97%, PI = 24.12%, a20-index = 16.13%, and IOA = 22.17% less than model PPV11. The percentage performance comparison of models PPV3, PPV8, and PPV9 demonstrates that model PPV9 obtained second place in achieving better performance. Model PPV9 has attained 1.42% less performance than model PPV11. Finally, the analysis introduces the LSTM-BA model PPV11 as the optimum performance model for predicting ground vibration during blasting.

Conversely, the sensitivity analysis demonstrates that H/B, B, S, and SD variables highly influence ground vibration prediction. Therefore, the obtained results in the testing phase have been analyzed for H/B, B, S, and SD variables.

#### Analysis of results for H/B

**Figure 14:**
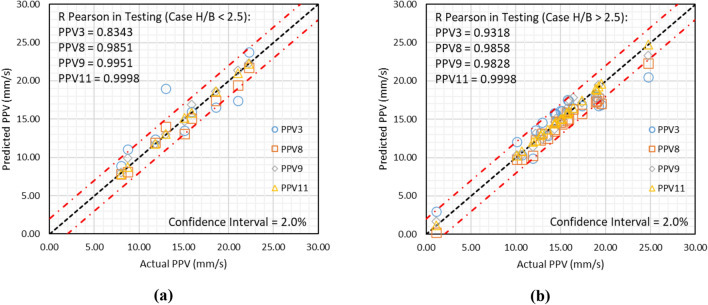
Depiction of results obtained using models PPV3, PPV8, PPV9, and PPV11, if (**a**) H/B < 2.5 and (**b**) H/B > 2.5.

The descriptive statistics demonstrate that the database contains H/B in the range of 0.476 to 3.833. Therefore, the prediction capabilities have been checked by bifurcating results for H/B greater than 2.5 and less than 2.5. Figure 14a and b presents the statistical relationship between actual and predicted PPV for H/B greater than 2.5 and less than 2.5.

Figure 14a illustrates that models PPV3, PPV8, PPV9, and PPV11 have predicted ground vibrations with R of 0.8343, 0.9851, 0.9951, and 0.9998, respectively, in the case of H/B is less than 2.5. Conversely, Fig. 14b demonstrates that models PPV3, PPV8, PPV9, and PPV11 have predicted ground vibrations with R of 0.9318, 0.9858, 0.9828, and 0.9998, respectively, in the case of H/B > 2.5. It is observed that (i) the GPR model PPV3 attains higher R if H/B > 2.5, and (ii) the LSTM-BA model PPV11 performs outstandingly in both cases (H/B < 2.5 and H/B > 2.5).

#### Analysis of results for B

**Figure 15:**
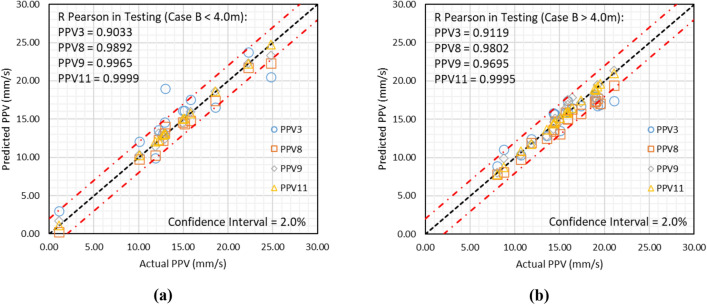
Depiction of results obtained using models PPV3, PPV8, PPV9, and PPV11, if (**a**) B < 4.0 m and (**b**) B > 4.0 m.

The database contains B in the range of 3.00–4.20 m. Therefore, the obtained results using models PPV3, PPV8, PPV9, and PPV11 have been analyzed for B less than 4.0 m and B higher than 4.0 m. The analyzed results for both cases are graphically depicted in Fig. 15a and b.

Figure 15 reveals that model PPV11 has predicted ground vibration with a performance of 0.9999 and 0.9995 for B < 4.0 m and B > 4.0, respectively. Figure 15a and b also demonstrates that the predicted ground vibrations using model PPV11 lie between the upper and lower confidence levels, and the confidence interval is 2.0%.

#### Analysis of results for S

**Figure 16:**
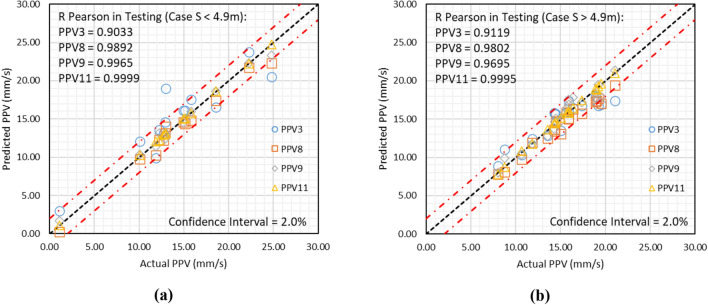
Depiction of results obtained using models PPV3, PPV8, PPV9, and PPV11, if (**a**) S < 4.9 m and (**b**) S > 4.9 m.

The database contains S in the range of 3.5–6.0 m. Therefore, the obtained results using models PPV3, PPV8, PPV9, and PPV11 have been analyzed for S less than 4.9 m and S higher than 4.9 m. The analyzed results for both cases are graphically depicted in Fig. 16a and b.

Figure 16a and b demonstrates that model PPV11 has attained 0.9999 and 0.9995 accuracies for S < 4.9 m and S > 4.9 m cases, respectively, comparatively higher than models PPV3, PPV8, and PPV9.

### Analysis of results SD

**Figure 17:**
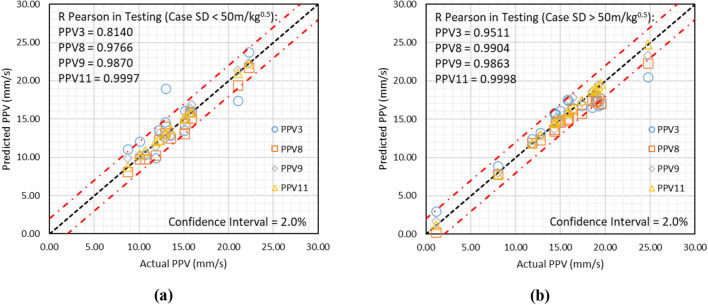
Illustration of results obtained using models PPV3, PPV8, PPV9, and PPV11, if (**a**) SD < 50 m/kg^0.5^ and (**b**) SD > 50 m/kg^0.5^.

The descriptive statistics show that the database contains scaling distance (SD) in the range of 16.6–160.420 m/kg^0.5^. Therefore, the testing results have been bifurcated for SD < 50 m/kg^0.5^ and SD > 50 m/kg^0.5^. A relationship between the actual and predicted ground vibrations has been drawn for each case, as depicted in Fig. 17.

Figure 17 shows that model PPV11 has outperformed other models PPV3, PPV8, and PPV9, with a performance of 0.9997 and 0.9998 in SD < 50 m/kg^0.5^ and SD > 50 m/kg^0.5^ cases, respectively.

The overall analysis of the results reveals that the LSTM-BA model PPV11 is highly capable of predicting ground vibrations (peak particle velocity-PPV) during blasting. Furthermore, the capabilities of the LSTM-BA model PPV11 in predicting the ground vibration have been validated by comparing the R of published models, as reported in Table 6. Table 6 illustrates that the LSTM-BA model PPV11 has outperformed the models available in the literature in predicting ground vibration during blasting. Hence, the LSTM-BA model PPV11 has been recognized as the optimum performance model.

**Table 6:** Validation of LSTM-BA model PPV11.

References	AI technique	Number of datapoints	Details of input variables		R Test
			Variables used	Nos	
Nguyen et al.^25^	SSO-ELM	216	B/S, DI, ST, MC, PF, HD, RQD, N, SD	9	0.9539
Ragam et al.^26^	XGBoost-RF	121	DI, ST, MC, PF, HD	5	0.9747
Zhang et al.^27^	RF	102	DI, MC	2	0.9695
Zhou et al.^28^	RF	102	DI, MC, PF, B, S, HD	6	0.9644
Huang et al.^29^	FA-ANN	88	DI, MC, B, S, N	5	0.9539
Zhou et al.^30^	GEP-MC	102	B/S, DI, ST, MC, PF, HD	6	0.9539
Nguyen et al.^11^	SVR-GA	125	DI, MC	2	0.9949
**Present study**	**LSTM-BA**	**162**	**N, B/De, H/B, B, S, Q, SD**	**7**	**0.9951**

*Bold values correspond to the optimum performance model.

### External validation

A model's generalizability is evaluated, and external validation is carried out to make sure the model isn't just overfitting the training set. Finding the most accurate model for predicting ground vibrations is made more accessible by the findings of external validation. Accuracy is the capacity of the model to correctly identify patients as having or not having the desired outcome. External validation checks for overfitting and guarantees that models are reliable. When a model is too tightly suited to the training data and does not generalize effectively to new data, it is said to be overfit. By contrasting the model's performance on the training data with the test data, external validation can help to spot overfitting. The Golbraikh and Tropsha^100^ theory, which was proposed, is an accurate model in this investigation. Table F (refer Appendix) summarizes the theory's various mathematical expression-related aspects. The external validation results are presented in Table 7 for all proposed models in both the training and testing phases. Table 7 demonstrates that model PPV11 has attained excellent generalizability, showing superiority over all PPV models employed in this work.

**Table 7:** Result obtained from external validation.

Model ID	Phase	$k$	$k{\prime}$	${R}_{o}^{2}$	${R{\prime}}_{o}^{2}$	${R}_{m}$	$\left|m\right|$	$\left|n\right|$
PPV1	TR	1.00	0.98	1.00	0.99	0.38	− 0.32	− 0.32
	TS	1.00	0.97	1.00	0.99	0.34	− 0.38	− 0.37
PPV2	TR	1.01	0.99	1.00	1.00	0.72	− 0.06	− 0.06
	TS	1.44	0.65	0.95	0.95	0.01	− 1.97	− 1.52
PPV3	TR	1.00	1.00	1.00	1.00	0.99	0.00	0.00
	TS	0.99	0.99	1.00	1.00	0.45	− 0.24	− 0.24
PPV4	TR	1.00	0.98	1.00	1.00	0.41	− 0.29	− 0.29
	TS	0.94	1.01	0.93	1.00	0.12	− 1.24	− 1.40
PPV5	TR	1.01	0.98	1.00	1.00	0.65	− 0.09	− 0.09
	TS	0.99	0.97	1.00	0.99	0.29	− 0.48	− 0.47
PPV6	TR	0.97	1.03	0.99	0.99	0.91	0.01	0.01
	TS	1.00	0.99	1.00	1.00	0.76	− 0.04	− 0.04
PPV7	TR	0.99	1.01	1.00	1.00	0.97	0.00	0.00
	TS	0.97	1.02	0.99	1.00	0.80	− 0.03	− 0.03
PPV8	TR	0.97	1.03	0.99	0.99	0.91	0.01	0.01
	TS	1.00	0.99	1.00	1.00	0.76	− 0.04	− 0.04
PPV9	TR	0.99	1.01	1.00	1.00	0.72	− 0.06	− 0.06
	TS	1.00	1.00	1.00	1.00	0.81	− 0.03	− 0.03
PPV10	TR	1.00	1.00	1.00	1.00	0.86	− 0.02	− 0.02
	TS	0.99	1.01	1.00	1.00	0.83	− 0.02	− 0.02
**PPV11**	**TR**	**1.00**	**1.00**	**1.00**	**1.00**	**0.98**	**0.00**	**0.00**
	**TS**	**1.00**	**1.00**	**1.00**	**1.00**	**0.98**	**0.00**	**0.00**

*Bold values present the optimum performance model.

### Overfitting analysis

**Figure 18:**
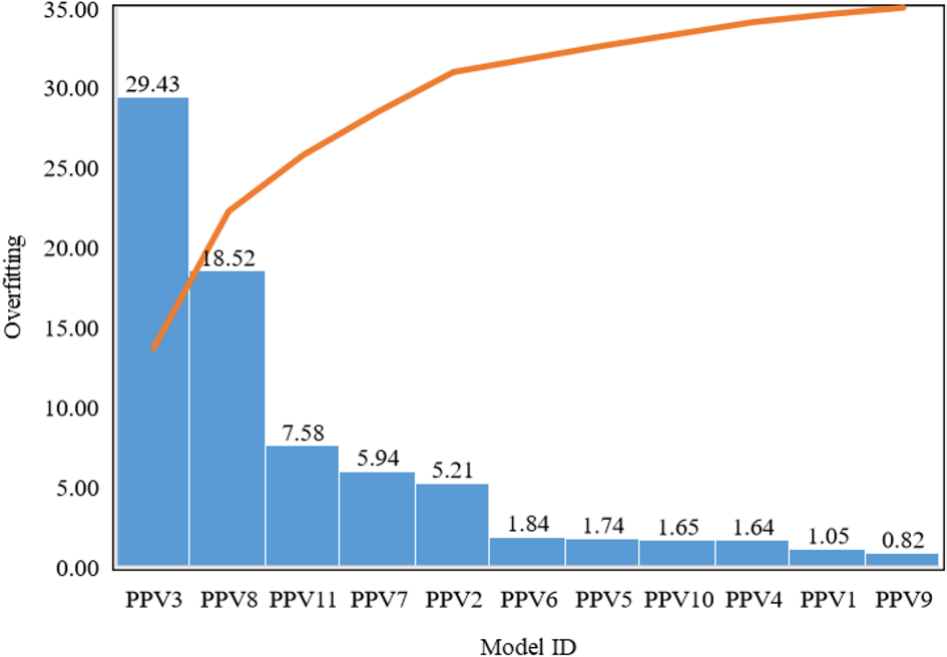
Depiction of overfitting achieved by models in predicting ground vibrations.

Overfitting is a phenomenon that occurs due to a complex model and a simple database or a simple model and a complex database. The ratio of test to train RMSE shows an overfitting of a computational model. In the present research, models PPV1 to PPV11 have been used to predict ground vibration during blasting. Overfitting of models PPV1 to PPV11 is estimated and depicted in Fig. 18.

Figure 18 demonstrates that the GPR model PPV3 has achieved the highest overfitting in predicting ground vibrations, followed by the G-LSSVM model PPV8. It has also been observed that model PPV11 has obtained 7.58 overfitting, comparatively less than models PPV3 and PPV8. The least overfitting has been attained by the ANN-BR model PPV9, i.e., 0.82.

## Summary and conclusions

This work introduces an optimum performance model for predicting ground vibration during blasting. For this aim, eleven computational models have been employed using machine, advanced machine, deep, and hybrid learning approaches. One hundred sixty-two data points have been collected from the field to develop, train, and test computational models. The present research also demonstrates the multicollinearity impact on the computational models. From the overall analysis, the following conclusions are drawn.

- The performance comparison reveals that the GPR (PPV3), Gaussian kernel function-based LSSVM (PPV8), Bayesian-regularization algorithm-based ANN (PPV9), and blackhole algorithm optimized LSTM (PPV11) models are highly capable of assessing the ground vibration during blasting. It is observed that the LSTM-BA model PPV11 outperformed the other models with high performance and accuracy. Also, the LSTM-BA model is quite easy to develop.
- The multicollinearity analysis shows that the burden (B) and spacing (S) variables have problematic multicollinearity. Moreover, the burden-to-diameter ratio (B/De), stiffness ratio (H/B), and scaling distance (SD) variables have considerable multicollinearity.
- The multicollinearity impact on the computational models has been studied in terms of accuracy and performance, and the following statements are drawn—(i) DT and RF did not perform well because the database contains moderate to problematic multicollinearity; (ii) Linear-LSSVM did not perform well because the complex database and model is too simple; (iii) GPR is less affected due to limited/ small database (large database may lead to problematic multicollinearity); (iv) LSTM did not achieve higher performance than ANN-BR because it requires large and good quality database; (v) Blackhole optimization technique improves the performance and accuracy of LSTM model in the multicollinearity.
- The following statements have been drawn in analyzing the overfitting of the model due to multicollinearity—(i) multicollinearity highly affects the overfitting of the covariance function-based model, i.e., GPR, (ii) multicollinearity highly affects the overfitting of the kernel function-based model, i.e., LSSVM, (iii) multicollinearity affect the overfitting of LSTM-BA model less than 10, (iv) LSTM-BA model has gained R = 1 in training phase presenting the overfitting wrt to R in the testing phase.
- The ANOVA and Z tests reject the null hypothesis for the present research. The Anderson–Darling test has also rejected the null hypothesis for predicted ground vibrations.
- The IOS, IOA, and a20-index are beneficial performance metrics to decide the optimum performance model. Based on the IOS, IOA, and a20-index, the LSTM-BA model PPV11 has been recognized as the optimum performance model.

To sum up, the present research introduces an optimum performance model LSTM-BA for predicting ground vibration during blasting. This work will help mining engineers/designers estimate the ground vibration during blasting. It is also suggested that the LSTM-BA model may be used to solve the different geotechnical issues. The Hunger Games Search, Reptile Search Algorithm, Artificial gorilla troops optimizer, Runge Kutta Optimizer, Dwarf Mongoose Optimization Algorithm, Artificial Hummingbird Algorithm, Aquila Optimizer, and Gradient-Based Optimizer may be used to optimize the LSTM approach. This is the first time that a common database has been used to employ the MLR, SVM, GPR, DT, ET, L-LSSVM, P-LSSVM, G-LSSVM, ANN-BR, LSTM, and LSTM-BA approaches in predicting of ground vibration during blasting.

### Supplementary Information

Supplementary Information.

## Data Availability

The datasets used and/or analysed during the current study available from the corresponding author on reasonable request.

## Abbreviations

ANFIS: Adaptive neuro fuzzy inference system
ANN-BR: Bayesian regularization based artificial neural network
ANOVA: Analysis of variance
AOC: Area over the curve
B: Burden
B/De: Burden-to-diameter ratio
B/S: Burden-to-spacing
BF: Bias factor
Bi: Blastability Index
CAM: Cosine amplitude method
CART: Classification and regression tree
CHIAD: Chi-square automatic interaction detection
D: Hole diameter
df: Degree of freedom
DI: Distance from the blast face
DPR: Delay per row
E: Young's modulus
ELM: Extreme learning machine
f: Rock hardness
F: F State
F Crit: F Critical
FA: Firefly algorithm
FIS: Fuzzy inference system
GEP: Gene expression programming
G-LSSVM: Gaussian kernel function-based LSSVM
H/B: Stiffness ratio
HD: Hole depth
HD/B: Hole depth-to-burden ratio
HKM: K-means clustering
HL: Hole length
ICA: Imperialist competitive algorithm
IOA: Index of agreement
IOS: Index of scatter
IQR: Interquartile range
Js: Joint spacing
L-LSSVM: Linear kernel function-based LSSVM
LMI: Legate and McCabe's Index
LSSVM: Least square support vector machine
LSTM: Long short-term memory
MAE: Mean absolute error
MAPE: Mean absolute percentage error
Max.: Maximum
MC: Maximum charge per delay
Min.: Minimum
MS: Means square
MSSD: Mean of the squared successive differences
N: Number of rows
n: Number of drilling holes
N: Number of datapoints
N*: Number of missing datapoints
NMBE: Normalized mean bias error
NS: Nash–Sutcliffe efficiency
PF: Powder factor
PI: Performance Index
P-LSSVM: Polynomial kernel function-based LSSVM
PPV: Peak particle velocity
PSO: Particle swarm optimization
Q: Explosive weight
Q1: First quartile
Q3: Third quartile
R: Correlation coefficient
R2: Coefficient of determination
RD: Rock density
REC: Regression error characteristics curve
RF: Random forest
RMR: Rock mass rating
RMSE: Root mean square error
ROC: Receiver operating characteristic curve
RQD: Rock quality designation
RSR: Root mean square error to observation's standard deviation ratio
S: Spacing
SC: Specific charge
SCA: Sine cosine algorithm
SD: Sub-drilling
SD: Scaling distance
SE: Square error
SS: Sum of squares
SSO: Sparrow search optimization
ST: Stemming
StDev: Standard deviation
SVM: Support vector machine
TC: Total charge
TS: Tensile strength
UCS: Uniaxial compressive strength
VAF: Variance accounted for
VIF: Variance inflation factor
Vp: P-Wave velocity
WMAPE: Weighted mean absolute percentage error
WOA: Whale optimization algorithm
XGBoost: Extreme gradient boosting
