# Supplementary material for: Assessment of the ground vibration during blasting in mining projects using different computational approaches
Source: Sci Rep. 2023 Oct 30;13:18582. doi: 10.1038/s41598-023-46064-5 (PMC10616075; doi:10.1038/s41598-023-46064-5)
Supplement: Supplementary file 1 — Supplementary Information. [file 41598_2023_46064_MOESM1_ESM.docx]

**Appendix**

Table A – Descriptive statistics of databases

| Variable | N | N* | Mean | SE Mean | StDev | Variance | CoefVar | Min. | Q1 | Median | Q3 | Max. | IQR | Skewness | Kurtosis | MSSD |
| --- | --- | --- | --- | --- | --- | --- | --- | --- | --- | --- | --- | --- | --- | --- | --- | --- |
| Overall Database | | | | | | | | | | | | | | | | |
| n | 162 | 0 | 77.280 | 3.540 | 45.050 | 2029.680 | 58.290 | 10.000 | 47.250 | 70.000 | 97.250 | 323.000 | 50.000 | 1.800 | 5.730 | 2160.960 |
| B/De | 162 | 0 | 0.006 | 0.000 | 0.003 | 0.000 | 41.510 | 0.003 | 0.004 | 0.006 | 0.007 | 0.014 | 0.003 | 1.050 | 0.430 | 0.000 |
| H/B | 162 | 0 | 2.463 | 0.047 | 0.603 | 0.364 | 24.480 | 0.476 | 2.619 | 2.691 | 2.750 | 3.833 | 0.131 | -1.880 | 2.820 | 0.378 |
| B (m) | 162 | 0 | 4.020 | 0.026 | 0.332 | 0.110 | 8.250 | 3.000 | 4.000 | 4.200 | 4.200 | 4.200 | 0.200 | -2.480 | 5.080 | 0.107 |
| S (m) | 162 | 0 | 4.846 | 0.024 | 0.307 | 0.094 | 6.340 | 3.500 | 4.800 | 5.000 | 5.000 | 6.000 | 0.200 | -1.890 | 5.610 | 0.088 |
| Q (kg/m^3^) | 162 | 0 | 1.315 | 0.133 | 1.690 | 2.857 | 128.570 | 0.229 | 0.675 | 0.792 | 0.994 | 12.082 | 0.319 | 3.760 | 15.390 | 2.874 |
| SD (m/kg^0.5^) | 162 | 0 | 57.600 | 1.820 | 23.110 | 534.090 | 40.120 | 16.600 | 39.450 | 55.490 | 73.790 | 160.420 | 34.340 | 0.770 | 1.430 | 502.640 |
| PPV (mm/s) | 162 | 0 | 15.077 | 0.342 | 4.354 | 18.956 | 28.880 | 1.135 | 12.470 | 15.042 | 17.749 | 28.197 | 5.279 | 0.080 | 0.880 | 19.972 |
| Training Database | | | | | | | | | | | | | | | | |
| n | 130 | 0 | 76.660 | 4.120 | 46.950 | 2204.190 | 61.240 | 10.000 | 44.000 | 65.000 | 95.000 | 323.000 | 51.000 | 1.930 | 6.110 | 2296.570 |
| B/De | 130 | 0 | 0.006 | 0.000 | 0.003 | 0.000 | 42.080 | 0.003 | 0.004 | 0.005 | 0.007 | 0.014 | 0.003 | 1.070 | 0.510 | 0.000 |
| H/B | 130 | 0 | 2.476 | 0.052 | 0.591 | 0.349 | 23.870 | 0.476 | 2.619 | 2.691 | 2.750 | 3.833 | 0.131 | -1.960 | 3.330 | 0.363 |
| B (m) | 130 | 0 | 4.019 | 0.029 | 0.329 | 0.108 | 8.190 | 3.000 | 4.000 | 4.200 | 4.200 | 4.200 | 0.200 | -2.490 | 5.200 | 0.113 |
| S (m) | 130 | 0 | 4.847 | 0.027 | 0.312 | 0.097 | 6.440 | 3.500 | 4.800 | 5.000 | 5.000 | 6.000 | 0.200 | -1.810 | 5.850 | 0.096 |
| Q (kg/m^3^) | 130 | 0 | 1.292 | 0.148 | 1.693 | 2.866 | 131.070 | 0.308 | 0.661 | 0.769 | 0.992 | 12.082 | 0.331 | 4.000 | 17.640 | 3.088 |
| SD (m/kg^0.5^) | 130 | 0 | 58.520 | 2.080 | 23.710 | 562.140 | 40.520 | 17.260 | 39.730 | 56.840 | 75.090 | 160.420 | 35.360 | 0.810 | 1.500 | 558.730 |
| PPV (mm/s) | 130 | 0 | 15.129 | 0.379 | 4.317 | 18.640 | 28.540 | 3.976 | 12.470 | 15.098 | 17.749 | 28.197 | 5.279 | 0.240 | 0.710 | 20.869 |
| Testing Database | | | | | | | | | | | | | | | | |
| n | 32 | 0 | 79.810 | 6.520 | 36.890 | 1360.740 | 46.220 | 20.000 | 50.000 | 78.500 | 99.500 | 178.000 | 49.500 | 0.660 | 0.420 | 1929.270 |
| B/De | 32 | 0 | 0.006 | 0.000 | 0.003 | 0.000 | 39.690 | 0.004 | 0.005 | 0.006 | 0.008 | 0.013 | 0.003 | 1.080 | 0.350 | 0.000 |
| H/B | 32 | 0 | 2.410 | 0.116 | 0.657 | 0.432 | 27.260 | 0.714 | 2.381 | 2.667 | 2.825 | 3.000 | 0.444 | -1.680 | 1.680 | 0.483 |
| B (m) | 32 | 0 | 4.025 | 0.061 | 0.347 | 0.121 | 8.630 | 3.000 | 4.000 | 4.200 | 4.200 | 4.200 | 0.200 | -2.570 | 5.640 | 0.086 |
| S (m) | 32 | 0 | 4.844 | 0.051 | 0.291 | 0.085 | 6.000 | 4.000 | 4.800 | 5.000 | 5.000 | 5.000 | 0.200 | -2.430 | 5.090 | 0.060 |
| Q (kg/m^3^) | 32 | 0 | 1.409 | 0.301 | 1.704 | 2.902 | 120.900 | 0.229 | 0.741 | 0.842 | 1.022 | 7.622 | 0.281 | 2.940 | 8.200 | 1.963 |
| SD (m/kg^0.5^) | 32 | 0 | 53.890 | 3.610 | 20.420 | 416.770 | 37.890 | 16.600 | 38.960 | 53.220 | 69.010 | 107.010 | 30.050 | 0.350 | 0.140 | 471.200 |
| PPV (mm/s) | 32 | 0 | 14.865 | 0.807 | 4.563 | 20.825 | 30.700 | 1.135 | 12.366 | 15.009 | 18.278 | 24.779 | 5.912 | -0.510 | 1.750 | 27.092 |

Table B – ANOVA analysis results for the whole database

| **ANOVA for** | ***Source of Variation*** | ***SS*** | ***df*** | ***MS*** | ***F*** | ***P-value*** | ***F crit*** |
| --- | --- | --- | --- | --- | --- | --- | --- |
| n | Between Groups | 313443.8 | 1 | 313443.8 | 306.002 | 1.25E-48 | 3.8705 |
|  | Within Groups | 329830.8 | 322 | 1024.319 |  |  |  |
|  | Total | 643274.6 | 323 |  |  |  |  |
| B/De | Between Groups | 18397.98 | 1 | 18397.98 | 1941.154 | 2.2E-138 | 3.8705 |
|  | Within Groups | 3051.87 | 322 | 9.477858 |  |  |  |
|  | Total | 21449.85 | 323 |  |  |  |  |
| H/B | Between Groups | 12888.88 | 1 | 12888.88 | 1334.302 | 1.5E-116 | 3.8705 |
|  | Within Groups | 3110.407 | 322 | 9.659649 |  |  |  |
|  | Total | 15999.29 | 323 |  |  |  |  |
| B | Between Groups | 9903.689 | 1 | 9903.689 | 1038.905 | 8.4E-103 | 3.8705 |
|  | Within Groups | 3069.566 | 322 | 9.532814 |  |  |  |
|  | Total | 12973.25 | 323 |  |  |  |  |
| S | Between Groups | 8478.43 | 1 | 8478.43 | 890.1233 | 1.07E-94 | 3.8705 |
|  | Within Groups | 3067.052 | 322 | 9.525006 |  |  |  |
|  | Total | 11545.48 | 323 |  |  |  |  |
| Q | Between Groups | 15341.96 | 1 | 15341.96 | 1406.697 | 1.5E-119 | 3.8705 |
|  | Within Groups | 3511.852 | 322 | 10.90637 |  |  |  |
|  | Total | 18853.81 | 323 |  |  |  |  |
| SD | Between Groups | 146494.9 | 1 | 146494.9 | 529.7782 | 5.4E-70 | 3.8705 |
|  | Within Groups | 89039.86 | 322 | 276.5213 |  |  |  |
|  | Total | 235534.8 | 323 |  |  |  |  |

Table C – Z-test results for the whole database

| ***Statistical parameters*** | ***n*** | ***B/De*** | ***H/B*** | ***B*** | ***S*** | ***Q*** | ***SD*** | ***PPV*** |
| --- | --- | --- | --- | --- | --- | --- | --- | --- |
| Mean | 77.28395 | 0.006212 | 2.46287 | 4.019753 | 4.846296 | 1.314711 | 57.60463 | 15.07723 |
| Known Variance | 2017.15 | 0.000007 | 0.36 | 0.11 | 0.09 | 2.84 | 530.79 | 18.84 |
| Observations | 162 | 162 | 162 | 162 | 162 | 162 | 162 | 162 |
| Hypothesized Mean Difference | 0 | 0 | 0 | 0 | 0 | 0 | 0 | - |
| z | 17.54717 | -44.1936 | -36.6414 | -32.3302 | -29.9294 | -37.6206 | 23.08825 | - |
| P(Z<=z) one-tail | 0 | 0 | 0 | 0 | 0 | 0 | 0 | - |
| z Critical one-tail | 1.644854 | 1.644854 | 1.644854 | 1.644854 | 1.644854 | 1.644854 | 1.644854 | - |
| P(Z<=z) two-tail | 0 | 0 | 0 | 0 | 0 | 0 | 0 | - |
| z Critical two-tail | 1.959964 | 1.959964 | 1.959964 | 1.959964 | 1.959964 | 1.959964 | 1.959964 | - |

Table D – Results obtained for soft computing models in the training and testing phase

| AI Approach | Model ID | Phase | RMSE | MAE | R | MAPE | VAF | WMAPE | NS | PI | BF | NMBE | LMI | RSR | a20 | IOA | IOS |
| --- | --- | --- | --- | --- | --- | --- | --- | --- | --- | --- | --- | --- | --- | --- | --- | --- | --- |
| MLR | PPV1 | Train | 0.0785 | 0.0616 | 0.8695 | 14.4002 | 75.60 | 0.1192 | 0.7560 | 1.4335 | 1.0286 | 0.0119 | 0.4977 | 0.4940 | 76.92 | 0.7511 | 0.1518 |
|  |  | Test | 0.0824 | 0.0669 | 0.8522 | 15.1534 | 72.55 | 0.1311 | 0.7249 | 1.3694 | 1.0278 | 0.0133 | 0.5625 | 0.5245 | 78.13 | 0.7187 | 0.1614 |
| SVM | PPV2 | Train | 0.0398 | 0.0312 | 0.9706 | 7.3009 | 93.73 | 0.0604 | 0.9373 | 1.8395 | 1.0147 | 0.0031 | 0.2522 | 0.2504 | 93.85 | 0.8739 | 0.0770 |
|  |  | Test | 0.2074 | 0.1774 | 0.7272 | 33.5117 | 51.97 | 0.3475 | 0.7433 | 0.8411 | 0.6666 | 0.0843 | 1.4908 | 1.3203 | 25.00 | 0.2546 | 0.4063 |
| GPR | PPV3 | Train | 0.0025 | 0.0020 | 0.9999 | 0.3893 | 99.98 | 0.0033 | 0.9998 | 1.9970 | 1.0009 | 0.0000 | 0.0137 | 0.0158 | 100.00 | 0.9932 | 0.0048 |
|  |  | Test | 0.0737 | 0.0580 | 0.8832 | 12.2871 | 78.00 | 0.1135 | 0.7797 | 1.4862 | 1.0187 | 0.0106 | 0.4871 | 0.4693 | 81.25 | 0.7564 | 0.1444 |
| DT | PPV4 | Train | 0.0755 | 0.0550 | 0.8801 | 13.3223 | 77.45 | 0.1063 | 0.7745 | 1.4736 | 1.0387 | 0.0110 | 0.4439 | 0.4748 | 85.38 | 0.7781 | 0.1459 |
|  |  | Test | 0.1241 | 0.0997 | 0.6588 | 27.7985 | 40.51 | 0.1953 | 0.3762 | 0.7150 | 1.1595 | 0.0302 | 0.8378 | 0.7898 | 65.63 | 0.5811 | 0.2431 |
| ET | PPV5 | Train | 0.0502 | 0.0349 | 0.9575 | 9.1946 | 90.05 | 0.0675 | 0.9004 | 1.7671 | 1.0380 | 0.0049 | 0.2818 | 0.3157 | 94.62 | 0.8591 | 0.0970 |
|  |  | Test | 0.0872 | 0.0602 | 0.8399 | 18.2228 | 69.35 | 0.1180 | 0.6922 | 1.3117 | 1.0972 | 0.0149 | 0.5063 | 0.5548 | 81.25 | 0.7469 | 0.1707 |
| L-LSSVM | PPV6 | Train | 0.0209 | 0.0180 | 0.9978 | 3.8342 | 99.54 | 0.0348 | 0.9826 | 1.9701 | 1.0383 | 0.0008 | 0.1455 | 0.1318 | 100.00 | 0.9273 | 0.0405 |
|  |  | Test | 0.0386 | 0.0305 | 0.9698 | 7.7061 | 93.98 | 0.0598 | 0.9398 | 1.8417 | 0.9937 | 0.0029 | 0.2567 | 0.2454 | 93.75 | 0.8717 | 0.0755 |
| P-LSSVM | PPV7 | Train | 0.0071 | 0.0061 | 0.9997 | 1.3227 | 99.94 | 0.0118 | 0.9980 | 1.9918 | 1.0131 | 0.0001 | 0.0491 | 0.0445 | 100.00 | 0.9754 | 0.0137 |
|  |  | Test | 0.0420 | 0.0346 | 0.9708 | 8.7486 | 94.01 | 0.0679 | 0.9285 | 1.8405 | 1.0355 | 0.0035 | 0.2911 | 0.2674 | 90.63 | 0.8545 | 0.0823 |
| G-LSSVM | PPV8 | Train | 0.0026 | 0.0021 | 0.9999 | 0.4924 | 99.99 | 0.0042 | 0.9997 | 1.9971 | 1.0047 | 0.0000 | 0.0173 | 0.0166 | 100.00 | 0.9913 | 0.0051 |
|  |  | Test | 0.0487 | 0.0382 | 0.9751 | 10.3971 | 95.07 | 0.0749 | 0.9038 | 1.8528 | 0.9054 | 0.0047 | 0.3214 | 0.3102 | 96.88 | 0.8393 | 0.0955 |
| ANN-BR | PPV9 | Train | 0.0379 | 0.0218 | 0.9719 | 4.9472 | 94.38 | 0.0422 | 0.9430 | 1.8504 | 1.0122 | 0.0028 | 0.1762 | 0.2386 | 96.15 | 0.9119 | 0.0733 |
|  |  | Test | 0.0311 | 0.0230 | 0.9809 | 6.4778 | 96.12 | 0.0451 | 0.9609 | 1.8924 | 0.9790 | 0.0019 | 0.1934 | 0.1977 | 96.88 | 0.9033 | 0.0608 |
| LSTM | PPV10 | Train | 0.0202 | 0.0175 | 0.9924 | 3.8017 | 98.39 | 0.0338 | 0.9839 | 1.9486 | 0.9995 | 0.0008 | 0.1410 | 0.1269 | 100.00 | 0.9295 | 0.0390 |
|  |  | Test | 0.0332 | 0.0264 | 0.9796 | 8.4018 | 95.52 | 0.0517 | 0.9552 | 1.8817 | 0.9759 | 0.0022 | 0.2217 | 0.2116 | 96.88 | 0.8891 | 0.0651 |
| LSTM-BA | **PPV11** | **Train** | **0.0024** | **0.0019** | **1.0000** | **0.3381** | **99.99** | **0.0032** | **0.9998** | **1.9972** | **1.0003** | **0.0000** | **0.0125** | **0.0151** | **100.00** | **0.9933** | **0.0046** |
|  |  | **Test** | **0.0181** | **0.0067** | **0.9951** | **3.8927** | **98.67** | **0.0131** | **0.9867** | **1.9587** | **0.9723** | **0.0006** | **0.0562** | **0.1155** | **96.88** | **0.9719** | **0.0356** |

***Bold value corresponds to an optimum performance model**

Table E – Results obtained from score analysis in the training and testing phase

| AI Approach | Model ID | Phase | RMSE | MAE | R | MAPE | VAF | WMAPE | NS | PI | BF | NMBE | LMI | RSR | a20 | IOA | IOS | Total | G-Total |
| --- | --- | --- | --- | --- | --- | --- | --- | --- | --- | --- | --- | --- | --- | --- | --- | --- | --- | --- | --- |
| MLR | PPV1 | Train | 1 | 1 | 1 | 1 | 1 | 1 | 1 | 1 | 4 | 1 | 1 | 1 | 1 | 1 | 1 | 18 | 72 |
|  |  | Test | 4 | 3 | 4 | 4 | 4 | 3 | 3 | 4 | 4 | 4 | 3 | 4 | 3 | 3 | 4 | 54 |  |
| SVM | PPV2 | Train | 4 | 4 | 4 | 4 | 4 | 4 | 4 | 4 | 5 | 4 | 4 | 4 | 3 | 4 | 4 | 60 | 91 |
|  |  | Test | 1 | 1 | 2 | 1 | 2 | 1 | 4 | 2 | 11 | 1 | 1 | 1 | 1 | 1 | 1 | 31 |  |
| GPR | PPV3 | Train | 10 | 10 | 9 | 10 | 9 | 10 | 10 | 9 | 9 | 10 | 10 | 10 | 6 | 10 | 10 | 142 | 216 |
|  |  | Test | 5 | 5 | 5 | 5 | 5 | 5 | 5 | 5 | 5 | 5 | 5 | 5 | 4 | 5 | 5 | 74 |  |
| DT | PPV4 | Train | 2 | 2 | 2 | 2 | 2 | 2 | 2 | 2 | 1 | 2 | 2 | 2 | 2 | 2 | 2 | 29 | 54 |
|  |  | Test | 2 | 2 | 1 | 2 | 1 | 2 | 1 | 1 | 1 | 2 | 2 | 2 | 2 | 2 | 2 | 25 |  |
| ET | PPV5 | Train | 3 | 3 | 3 | 3 | 3 | 3 | 3 | 3 | 3 | 3 | 3 | 3 | 4 | 3 | 3 | 46 | 94 |
|  |  | Test | 3 | 4 | 3 | 3 | 3 | 4 | 2 | 3 | 2 | 3 | 4 | 3 | 4 | 4 | 3 | 48 |  |
| L-LSSVM | PPV6 | Train | 6 | 6 | 7 | 6 | 7 | 6 | 6 | 7 | 2 | 6 | 6 | 6 | 6 | 6 | 6 | 89 | 202 |
|  |  | Test | 8 | 8 | 6 | 9 | 6 | 8 | 8 | 7 | 6 | 8 | 8 | 8 | 7 | 8 | 8 | 113 |  |
| P-LSSVM | PPV7 | Train | 8 | 8 | 8 | 8 | 8 | 8 | 8 | 8 | 6 | 8 | 8 | 8 | 6 | 8 | 8 | 116 | 215 |
|  |  | Test | 7 | 7 | 7 | 7 | 7 | 7 | 7 | 6 | 3 | 7 | 7 | 7 | 6 | 7 | 7 | 99 |  |
| G-LSSVM | PPV8 | Train | 9 | 9 | 10 | 9 | 10 | 9 | 9 | 10 | 8 | 9 | 9 | 9 | 6 | 9 | 9 | 134 | 236 |
|  |  | Test | 6 | 6 | 8 | 6 | 8 | 6 | 6 | 8 | 10 | 6 | 6 | 6 | 8 | 6 | 6 | 102 |  |
| ANN-BR | PPV9 | Train | 5 | 5 | 5 | 5 | 5 | 5 | 5 | 5 | 7 | 5 | 5 | 5 | 5 | 5 | 5 | 77 | 222 |
|  |  | Test | 10 | 10 | 10 | 10 | 10 | 10 | 10 | 10 | 7 | 10 | 10 | 10 | 8 | 10 | 10 | 145 |  |
| LSTM | PPV10 | Train | 7 | 7 | 6 | 7 | 6 | 7 | 7 | 6 | 11 | 7 | 7 | 7 | 6 | 7 | 7 | 105 | 237 |
|  |  | Test | 9 | 9 | 9 | 8 | 9 | 9 | 9 | 9 | 8 | 9 | 9 | 9 | 8 | 9 | 9 | 132 |  |
| LSTM-BA | **PPV11** | **Train** | **11** | **11** | **11** | **11** | **11** | **11** | **11** | **11** | **10** | **11** | **11** | **11** | **6** | **11** | **11** | **159** | **319** |
|  |  | **Test** | **11** | **11** | **11** | **11** | **11** | **11** | **11** | **11** | **9** | **11** | **11** | **11** | **8** | **11** | **11** | **160** |  |

***Bold value corresponds to the optimum performance model**

Table F – Mathematical expression and condition of validation factors

| Validation Parameters | Condition | Mathematical Expression |
| --- | --- | --- |
| $\boldsymbol{k}$ | $0.85<k<1.15$ | $k=\frac{\sum_{i=0}^{n} (d_{i}\times y_{i})}{\sum_{i=0}^{n} y_{i}^{2}}$ |
| $\boldsymbol{k'}$ | $0.85<k'<1.15$ | $k'=\frac{\sum_{i=0}^{n} (d_{i}\times y_{i})}{\sum_{i=0}^{n} d_{i}^{2}}$ |
| $\boldsymbol{R}_{\boldsymbol{o}}^{\boldsymbol{2}}$ | Close to 1 | $R_{o}^{2}=1-\frac{\sum_{i=1}^{n} y_{i}^{2}\left( 1-k \right)^{2}}{\sum_{i=1}^{n} (y_{i}-\bar{y})}$ |
| $\boldsymbol{R'}_{\boldsymbol{o}}^{\boldsymbol{2}}$ | Close to 1 | ${R'}_{o}^{2}=1-\frac{\sum_{i=1}^{n} d_{i}^{2}\left( 1-k' \right)^{2}}{\sum_{i=1}^{n} (d_{i}-\bar{d})}$ |
| $\boldsymbol{R}_{\boldsymbol{m}}$ | $R_{m}>0.5$ | $R_{m}=R^{2}\times\left( 1-\sqrt{\left\vert R^{2}-R_{o}^{2} \right\vert} \right)$ |
| $\left\vert\boldsymbol{m} \right\vert$ | $\left\vert m \right\vert<0.1$ | $m=\frac{R^{2}-R_{o}^{2}}{R^{2}}$ |
| $\left\vert\boldsymbol{n} \right\vert$ | $\left\vert n \right\vert<0.1$ | $n=\frac{R^{2}-R_{o}^{'2}}{R^{2}}$ |

Where $d_{i}$ denotes the experimental pile capacity and $y_{i}$ denotes the predicted pile capacity, $k$ and $k'$ represent the slopes of the predicted versus actual pile capacity and actual versus predicted pile capacity with respect to the origin. $R_{o}^{2}$ and ${R'}_{o}^{2}$ denotes the coefficients of determination of the predicted versus actual pile capacity and actual versus predicted pile capacity. $m$ and $n$ represent the factors for estimating the predictive power of the proposed models.
